# Aldehyde dehydrogenase 2 and PARP1 interaction modulates hepatic HDL biogenesis by LXR**α**-mediated ABCA1 expression

**DOI:** 10.1172/jci.insight.155869

**Published:** 2022-04-08

**Authors:** Luxiao Li, Shanshan Zhong, Rui Li, Ningning Liang, Lili Zhang, Shen Xia, Xiaodong Xu, Xin Chen, Shiting Chen, Yongzhen Tao, Huiyong Yin

**Affiliations:** 1 CAS Key Laboratory of Nutrition, Metabolism and Food Safety, Shanghai Institute of Nutrition and Health (SINH), University of the Chinese Academy of Sciences (UCAS), Chinese Academy of Sciences (CAS), Shanghai, China.; 2 School of Life Science and Technology, ShanghaiTech University, Shanghai, China.; 3 Key Laboratory of Food Safety Risk Assessment, Ministry of Health, Beijing, China.

**Keywords:** Cell Biology, Metabolism, Cardiovascular disease, Cholesterol, Lipoproteins

## Abstract

HDL cholesterol (HDL-C) predicts risk of cardiovascular disease (CVD), but the factors regulating HDL are incompletely understood. Emerging data link CVD risk to decreased HDL-C in 8% of the world population and 40% of East Asians who carry an SNP of aldehyde dehydrogenase 2 (ALDH2) rs671, responsible for alcohol flushing syndrome; however, the underlying mechanisms remain unknown. We found significantly decreased HDL-C with increased hepatosteatosis in ALDH2-KO (AKO), ALDH2/LDLR–double KO (ALKO), and ALDH2 rs671–knock-in (KI) mice after consumption of a Western diet. Metabolomics identified ADP-ribose as the most significantly increased metabolites in the ALKO mouse liver. Moreover, ALDH2 interacted with poly(ADP-ribose) polymerase 1 (PARP1) and attenuated PARP1 nuclear translocation to downregulate poly(ADP-ribosyl)ation of liver X receptor α (LXRα), leading to an upregulation of ATP-binding cassette transporter A1 (ABCA1) and HDL biogenesis. Conversely, AKO or ALKO mice exhibited lower HDL-C with ABCA1 downregulation due to increased nuclear PARP1 and upregulation of LXRα poly(ADP-ribosyl)ation. Consistently, PARP1 inhibition rescued ALDH2 deficiency–induced fatty liver and elevated HDL-C in AKO mice. Interestingly, KI mouse or human liver tissues showed ABCA1 downregulation with increased nuclear PARP1 and LXRα poly(ADP-ribosyl)ation. Our study uncovered a key role of ALDH2 in HDL biogenesis through the LXRα/PARP1/ABCA1 axis, highlighting a potential therapeutic strategy in CVD.

## Graphical abstract

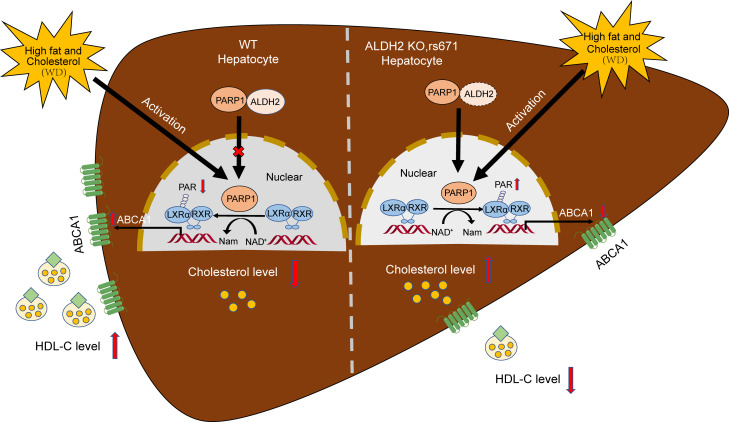

## Introduction

Cardiovascular disease (CVD) has remained the leading cause of death worldwide for decades, and atherosclerosis is one of the main underlying conditions for developing CVD (1–3). Atherosclerosis is closely associated with dyslipidemia, oxidative stress, and chronic inflammation, among which dysregulation of cholesterol metabolism plays an important role (4, 5). It is well-established that elevated levels of circulating LDL cholesterol (LDL-C) and low levels of HDL cholesterol (HDL-C) are important risk factors for developing atherosclerosis and CVD (6, 7). Thus, cholesterol-lowering therapies, such as statins or PCSK9 antibodies, have been clinically used for the prevention and treatment of CVD (2, 8). Although the mechanisms underlying the general protective role of HDL in CVD remain to be clearly defined, raising HDL has been extensively explored as an attractive therapeutic strategy, especially for targeting residual risk factors of CVD (9–12). HDL biogenesis is a complex process that involves the synthesis and secretion of the major protein components of HDL, such as ApoA-I (13, 14). Liver-synthesized ApoA-I quickly acquires cholesterol via the hepatocyte ATP-binding cassette transporter A1 (ABCA1) (15, 16). Liver X receptors (LXRs), a member of the nuclear receptor family, play a pivotal role in lipid metabolism, and emerging evidence suggests that LXRs also participate in HDL biogenesis by regulating ABCA1 expression (17).

Poly(ADP-ribose) polymerase1 (PARP1) is a key DNA damage repairing enzyme and plays an important role in cell stress response, which synthesizes ADP-ribose units by NAD (18, 19). PARP1 also participates in the posttranslational modification of many proteins and plays an important role in the regulation of cancer, inflammation, and metabolic diseases (20, 21). Emerging studies identified an important role of LXRα and PARP1 in cholesterol metabolism, including cholesterol efflux in macrophages (22) and cholesterol deposition in the liver (23).

Aldehyde dehydrogenase (ALDH) is an enzyme family dependent on NAD(P)^+^ with 19 members of isozymes, among which ALDH2 is primarily localized in mitochondria in various tissues and responsible for catalyzing endogenous and exogenous aldehydes oxidation (24). A major SNP of the human ALDH2 gene (ALDH2 rs671) results in amino acid substitution of Glu504Lys, which affects 8% of the world population and around 40% of East Asians. In addition to alcohol flushing due to the increased acetaldehyde levels as a result of significantly decreased enzyme activity, this SNP has been associated with increased risk of CVD, but the underlying mechanisms remain poorly defined (25–28). Although previous studies have been largely focused on the enzymatic activity of ALDH2 in the pathogenesis of CVD (25), emerging evidence indicates diverse mechanisms beyond the alcohol consumption (27–29). Recent human data have identified that ALDH2 rs671 is among the novel East Asian–specific coding variants that contribute to lipid levels, including LDL-C and HDL-C, and CVD (30, 31). However, the underlying molecular mechanisms linking ALDHs to cholesterol metabolism await further investigation. We identified a potentially novel role of ALDH2 in macrophage foam cell formation through interactions with LDL receptor (LDLR) and AMPK (29), linking ALDH2 SNP with increased risks of CVD beyond alcohol consumption (32). Further study showed that hepatic ALDH2 regulates the protein stability of HMG-CoA reductase (HMGCR), the rate-limiting enzyme of cholesterol de novo synthesis and target of statins; ALDH2 rs671 variant stabilizes HMGCR and promotes cholesterol synthesis, which leads to higher levels of total cholesterol in mice and humans (29, 33).

In this study, we aimed to investigate the roles and mechanisms of ALDH2 in HDL biogenesis using ALDH2-KO (AKO) and rs671–knock-in (rs671-KI) mice as well as human clinical samples. ALDH2 deficiency in AKO or double KO of ALDH2 and LDLR (ALKO) mice led to decreased HDL-C levels and enhanced hepatosteatosis after feeding with a Western diet (WD), similar to ALDH2 rs671-KI mice. Untargeted metabolomics uncovered an unexpected accumulation of ADP-ribose in the liver tissues, and further mechanistic studies provided evidence that ALDH2 modulates the nuclear translocation of PARP1 and poly(ADP-ribosyl)ation of LXRα, which regulates hepatic ABCA1 expression and HDL biogenesis. Consistently, pharmacological inhibition of PARP1 rescued decreased HDL-C levels through upregulating hepatic ABCA1 and ApoA-I expressions in AKO mice with WD feeding and attenuated hepatosteatosis. Our study has uncovered a mechanism in HDL biogenesis by which ALDH2 interacts with PARP1 through the LXRα/PARP1/ABCA1 axis.

## Results

### ALDH2 deficiency in ALDH2-KO or ALDH2/LDLR-double KO mice results in decreased levels of circulating HDL but aggravated hepatosteatosis with WD feeding.

**Figure 1:**
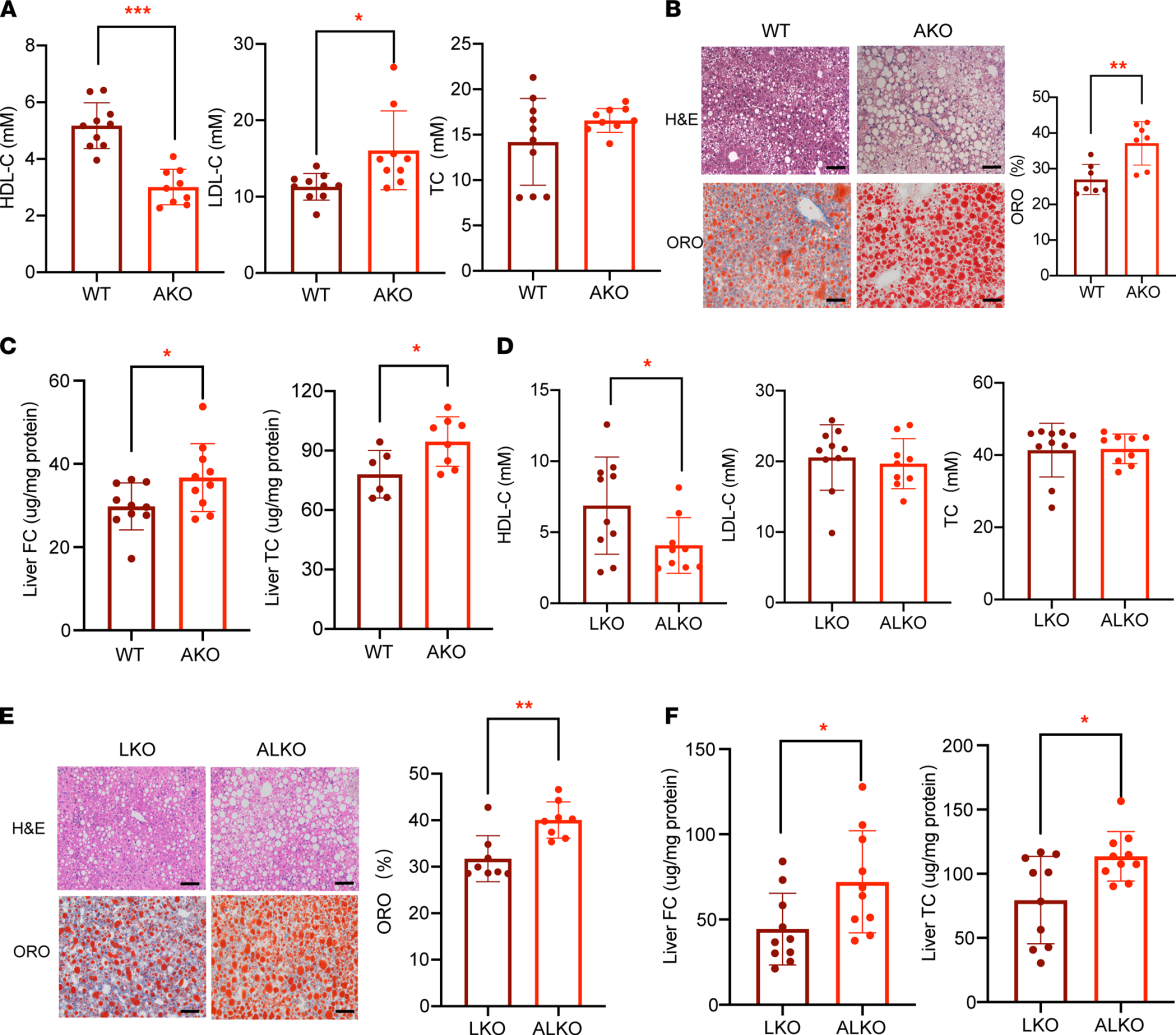
ALDH2 deficiency exhibits lower levels of circulating HDL-C but increased total cholesterol in the mouse liver with a Western diet. (**A**) TC, LDL-C, and HDL-C in WT and AKO mouse plasma at 32^nd^ week (Western diet [WD] for 26 weeks. WT, *n* = 10; AKO, *n* = 9. (**B**) Representative H&E and Oil Red O staining for the mouse liver tissues at 32^nd^ week (scale bar: 100 μm). Quantification of lipid droplet areas of liver from WT and AKO mice. WT, *n* = 7; AKO, *n* = 7. (**C**) TC (WT, *n* = 10; AKO, *n* = 10) and FC (WT, *n* = 6; AKO, *n* = 8) levels in WT and AKO liver. (**D**) TC, LDL-C, and HDL-C levels in LKO and ALKO mouse plasma at 32^nd^ week (WD for 26 weeks). LKO, *n* = 10; ALKO, *n* = 9. (**E**) Representative H&E and Oil Red O staining for mouse liver tissues (scale bar: 100 μm). Quantification of lipid droplet areas of liver from LKO and ALKO mice. LKO, *n* = 8; ALKO, *n* = 8. (**F**) TC and FC in LKO and ALKO liver tissues. LKO, *n* = 10; ALKO, *n* = 10. Statistical comparisons were made using a 2-tailed Student’s *t* test. All data are mean ± SD. *P < 0.05, **P < 0.01, ***P < 0.001. AKO, ALDH2-KO; TC, total cholesterol; FC, free cholesterol; LKO, LDLR KO; ALKO, ALDH2/ALDH2-KO.

To determine the roles of ALDH2 in cholesterol metabolism in the context of atherosclerosis, we generated ALDH2-KO (AKO) and ALDH2/LDLR-double KO (ALKO) mice and fed them and their controls with a WD or chow diet (CD) for 26 weeks. Interestingly, CD feeding did not significantly change the HDL-C levels and body weights between AKO and WT mice but significantly promoted hepatic steatosis in AKO mice (Supplemental Figure 1, A–C; supplemental material available online with this article; https://doi.org/10.1172/jci.insight.155869DS1), presumably through regulating de novo cholesterol biosynthesis and HMGCR protein stability (33) (see complete unedited blots in the supplemental material). Moreover, CD feeding did not alter plasma HDL-C levels, hepatic steatosis, or body weights between LKO and ALKO mice (Supplemental Figure 1, D–F). However, WD feeding significantly decreased levels of plasma HDL-C but increased levels of LDL-C in AKO mice, whereas the levels of total cholesterol remained similar between the AKO and WT groups (Figure 1A). Furthermore, WD feeding led to increased hepatic steatosis and elevated levels of free cholesterol and total cholesterol in the liver tissues of AKO compared with WT mice (Figure 1, B and C). Most of these phenotypes were recapitulated in WD-fed ALDH2/LDLR-KO (ALKO) mice compared with LDLR-KO (LKO) mice (Figure 1, D–F), except that the LDL-C levels in the plasma remained similar between these 2 groups with WD feeding (Figure 1D). Taken together, all these data suggest that ALDH2 plays an important role in modulating circulating HDL levels and hepatic cholesterol HDL biogenesis.

### ALDH2 deficiency decreases hepatic ABCA1 expression in mice when fed with WD.

**Figure 2:**
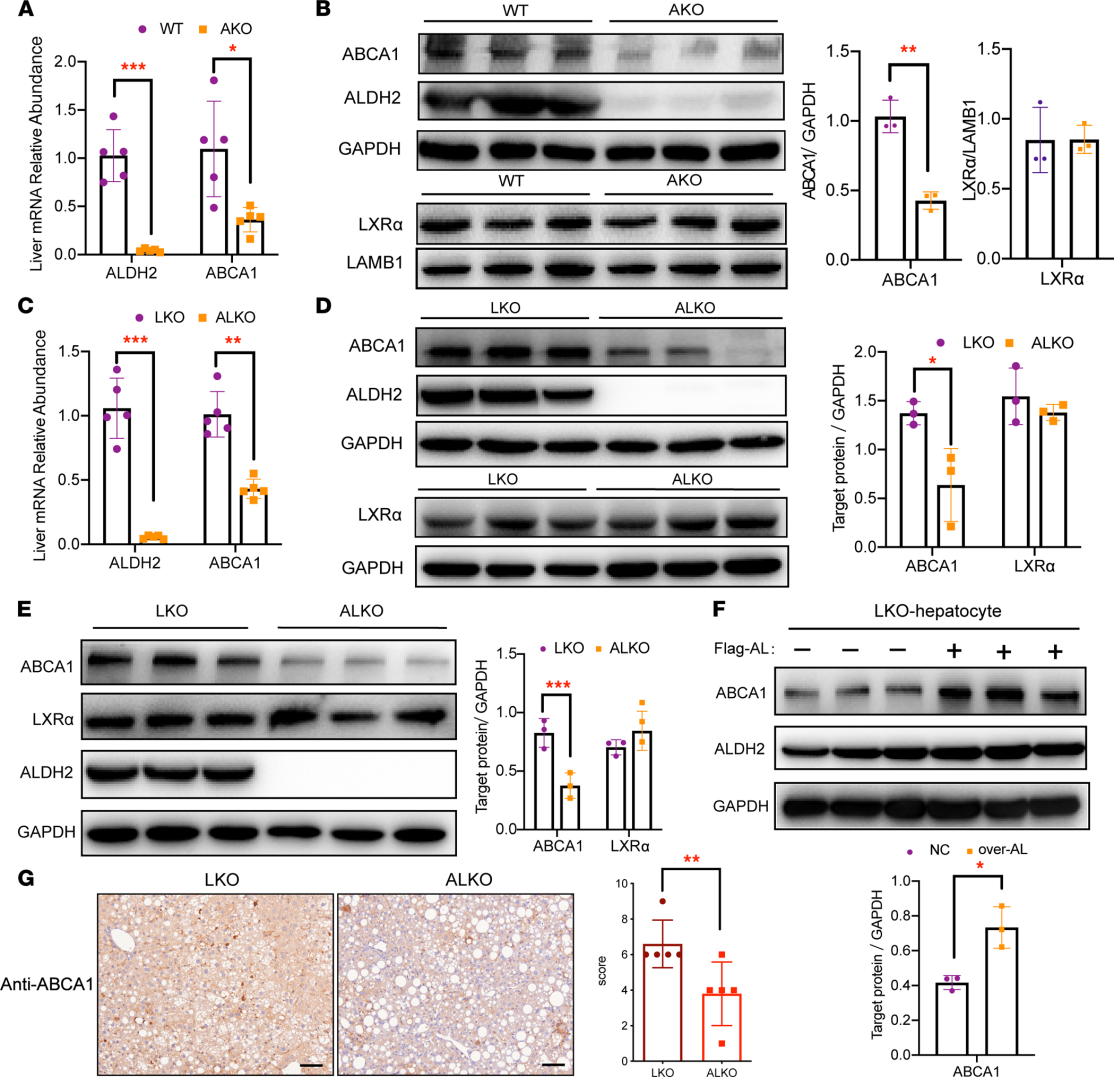
ALDH2 deficiency and Western diet feeding decreases hepatic ABCA1 expression without significantly affecting LXRα. (**A**) Hepatic mRNA levels of ABCA1 and ALDH2 in WT and AKO mouse tissues at 32^nd^ week (Western diet for 26 weeks). WT, *n* = 5; AKO, *n* = 5. (**Β**) Western blotting analysis of ABCA1 and LXRα expressions in WT and AKO liver tissue. WT, *n* = 3; AKO, *n* = 3. (**C**) Hepatic mRNA levels of LXRα and ABCA1 in LKO and ALKO mice at 32nd week (Western diet for 26 weeks). LKO, *n* = 5; ALKO, *n* = 5. (**D**) Western blotting analysis of ABCA1 and LXRα expression in LKO and ALKO liver tissues. LKO, *n* = 3; ALKO, *n* = 3. (**E**) Western blotting analysis of ABCA1; LXRα expression in LKO and ALKO hepatocytes treated with ox-LDL (50 μg/mL, 16 h). LKO, *n* = 3; ALKO, *n* = 3. (**F**) Western blotting analysis of ABCA1 expression in overexpressed ALDH2 (over-AL) hepatocytes treated with ox-LDL (50 μg/mL, 16 h). LKO, *n* = 3; ALKO, *n* = 3. (**G**) IHC analysis of ABCA1 expressions in mouse liver sections (LKO, *n* = 5; ALKO, *n* = 5; scale bar: 100 μm). Statistical comparisons were made using a 2-tailed Student’s *t* test. All data are mean ± SD. *P < 0.05, **P < 0.01, ***P < 0.001.

The observation that ALDH2 deficiency and WD feeding in mice resulted in decreased plasma HDL levels but increased cholesterol and steatosis in the liver prompted us to hypothesize that ALDH2 plays an important role in hepatic HDL biogenesis by modulating the expression or activities of cholesterol transporters, such as ABCA1. Consistently, we found that mRNA levels of ABCA1 in WD-fed AKO mouse liver were significantly downregulated compared with WT mice (Figure 2A). Interestingly, the protein levels of ABCA1 in these mice were also significantly downregulated, but LXRα and SR-B1 were not (Figure 2B and Supplemental Figure 2A). Next, we detected the mRNA levels of genes associated with cholesterol metabolic homeostasis, including biosynthesis (*Hmgcr*, *Hmgcs,* and *Srebp2*), transport (*Sr-b1*, *Abca1*, and *Abca9*), and secretion (*Abcg5*, *Abcg8,* and *Cyp7a1*) in the liver of WD-fed ALKO and LKO mice, and we found that the mRNA level of *Abca1* significantly decreased and *Sr-b1* significantly increased in ALKO liver tissues compared with LKO mice (Figure 2C and Supplemental Figure 2B). The protein expression of ABCA1 was significantly decreased and SR-B1 was slightly increased, whereas the protein levels of ABCG5 and CYP7A1 did not significantly change in ALKO liver tissue (Figure 2D and Supplemental Figure 2C). Notably, CD feeding did not significantly affect ABCA1 expression in WT and AKO mice (Supplemental Figure 2F).

The fact that ALDH2-KO inhibits ABCA1 expressions without affecting LXRα, an upstream factor regulating ABCA1 and SR-B1 expression, led us to hypothesize that posttranslation regulation of LXRα may play a more important role in regulating ABCA1 expression and circulating HDL. To this end, we examined the effects of ALDH2 on ABCA1 expression in mouse primary hepatocytes. As shown in Figure 2E, the protein levels of ABCA1 significantly decreased in oxidized low-density lipoprotein–treated (ox-LDL–treated) ALKO hepatocytes compared with LKO hepatocytes. Conversely, ox-LDL treatment of LKO hepatocytes overexpressing ALDH2 led to an upregulation of ABCA1, consistent with the observation that CD feeding did not significantly affect ABCA1 expression in LKO and ALKO mice (Figure 2F and Supplemental Figure 2, D and E). More importantly, tissue IHC staining of liver sections from ALKO mice displayed a 50% decrease in ABCA1-positive cells versus those from LKO mice (Figure 2G). Taken together, ALDH2 deficiency and WD feeding decreased mouse plasma HDL but increased cholesterol levels of liver tissue, primarily because of the downregulation of ABCA1 expression in the liver.

### Untargeted metabolomics identifies ADP-ribose and purine metabolic pathways as the most significantly altered metabolites and pathways in the liver of ALKO mice compared with LKO mice after WD feeding.

**Figure 3:**
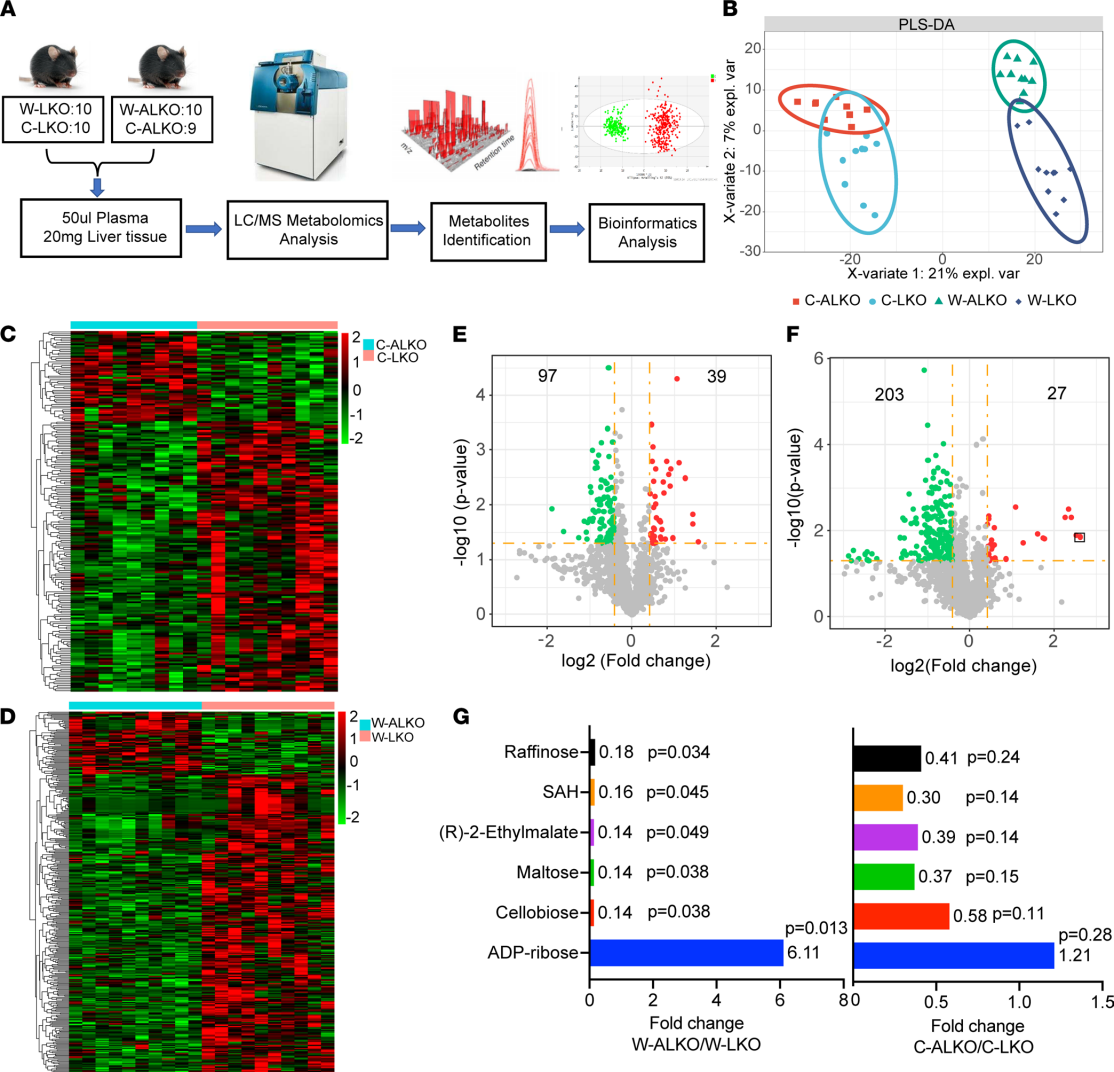
Untargeted metabolomics identifies ADP-ribose as the most significantly differentiated metabolites between ALKO and LKO mouse liver tissue with WD feeding. (**A**) Study design and metabolomics workflow. (**B**) Partial least squares discriminant analysis (PLS-DA). (**C** and **D**) The heatmap of differential metabolites in mouse liver tissue at 32^nd^ week with chow diet and Western diet (WD), respectively. (**E**) The volcano plot of differential metabolites in LKO and ALKO liver tissue with chow diet. Red dots represent significantly upregulated metabolites, *P* < 0.05; green dots represent significantly downregulated metabolites, *P* < 0.05; the yellow dotted line indicates fold change > 4/3 or fold change < 3/4. (**F**) The volcano plot of differential metabolites in LKO and ALKO liver tissue with WD for 26 weeks. Black boxed metabolite was ADP-ribose. (**G**) Significantly changed metabolites: fold change > 5, fold change < 1/5. W-LKO, *n* = 10; W-ALKO, *n* = 10; C-LKO, *n* = 9; C-ALKO, *n* = 9. Statistical comparisons were made using a 2-tailed Student’s *t* test. All data are mean ± SD. “C” in front of LKO or ALKO indicates chow diet and “W” indicates Western diet.

To explore the molecular mechanisms that may be potentially responsible for the downregulation of ABCA1 expression in ALDH2-KO mouse liver tissues, we performed untargeted metabolomics on mouse plasma and liver tissues using a high-resolution mass spectrometry–based metabolomics approach previously established in our laboratory (34). Four groups of mice were used in our metabolomic analyses based on the workflow in Figure 3A. We detected 46,108 metabolic peaks in positive ionization modes and 31,180 metabolic peaks in negative ionization modes in liver tissues and identified 2599 metabolites after the database search. The supervised orthogonal partial least squares discriminant analysis (PLS-DA) of metabolites in mouse liver tissues showed a better separation of all metabolites in LKO and ALKO mice fed with WD compared with mice fed with CD (Figure 3B). Consistently, hierarchical clustering analyses were utilized to explore the global metabolic variations among each group (Figure 3, C and D). Next, we observed 136 statistically significant differential metabolites with fold change greater than 4/3 or less than 3/4 in the CD group, among which 39 metabolites were upregulated and 97 metabolites were downregulated in LKO liver tissues compared with ALKO liver tissue (Figure 3E). For WD groups, we detected 230 statistically significant differential metabolites with fold change greater than 4/3 or less than 3/4, among which 27 metabolites were upregulated and 203 metabolites were downregulated in LKO liver tissue compared with ALKO liver tissue (Figure 3F). These results suggested that WD feeding led to more pronounced metabolic alterations than those for CD. More importantly, we found that ADP-ribose ranked at the top of these significantly differential metabolites in the WD group (Figure 3G and Supplemental Figure 3B). Notably, none of these differential metabolites had a significant change in the CD group. Next, we performed a pathway enrichment analysis with these significantly different metabolites with *P* less than 0.05. In the CD group, pathway enrichment analysis demonstrated that significantly differential metabolites belonged to 13 pathways, and the top 3 pathways were galactose metabolism, fructose and mannose metabolism, and arginine and proline metabolism (Supplemental Figure 3A). In the WD group, pathway enrichment analysis demonstrated that significantly differential metabolites belonged to 16 pathways, and the top 3 pathways were galactose metabolism, fructose and mannose metabolism, and purine metabolism (Supplemental Figure 3, A and B), suggesting a potential mechanistic link between the purine metabolism pathway and ALDH2-mediated ABCA1 expression and HDL biogenesis in the mouse liver with WD feeding. We performed similar metabolomics analyses on the plasma samples from these 4 groups (Supplemental Figure 3, C–F). However, ADP-ribose was not significantly changed in plasma samples, although the purine pathway was also ranked at the top with WD feeding in the pathway enrichment analysis (Supplemental Figure 3, F and G). Overall, ADP-ribose and the purine pathway were significantly upregulated in the liver tissue of ALKO mice compared with LKO mice, which may be responsible for ALDH2-regulated HDL biogenesis.

### ALDH2 and PARP1 interaction attenuates nuclear translocation of PARP1, resulting in decreased poly(ADP-ribosyl)ation of LXRα and upregulation of ABCA1 expression.

PARP1 mainly uses NAD^+^ as a substrate to covalently modify Glu, Asp, or Lys residues of the receptor proteins for ribosylation modification to form PAR, which represents one of the common posttranslational modification of various proteins, whereas poly-ADP-ribose glycohydrolase (PARG) cleaves PAR to form free ADP-ribose and turns off ribosylation signaling (18). The significant increase of ADP-ribose in liver tissues of ALKO compared with LKO mice prompted us to hypothesize that PARP1 and downstream signaling are important in regulating hepatic ABCA1 expression and HDL biogenesis. To test whether a similar mechanism operates in hepatocytes in regulating HDL levels, we found that poly(ADP-ribosyl)ation of proteins was increased in liver tissues of ALKO compared with LKO mice (Supplemental Figure 4A). Conversely, overexpressing ALDH2 in ox-LDL–treated LKO hepatocytes led to decreased levels of poly(ADP-ribosyl)ation of proteins (Supplemental Figure 4B). Interestingly, there was no significant difference in poly(ADP-ribosyl)ation in the LKO and ALKO liver with CD feeding (Supplemental Figure 4C). These results demonstrated that ALDH2 affected hepatic PARP1 activity with WD feeding.

**Figure 4:**
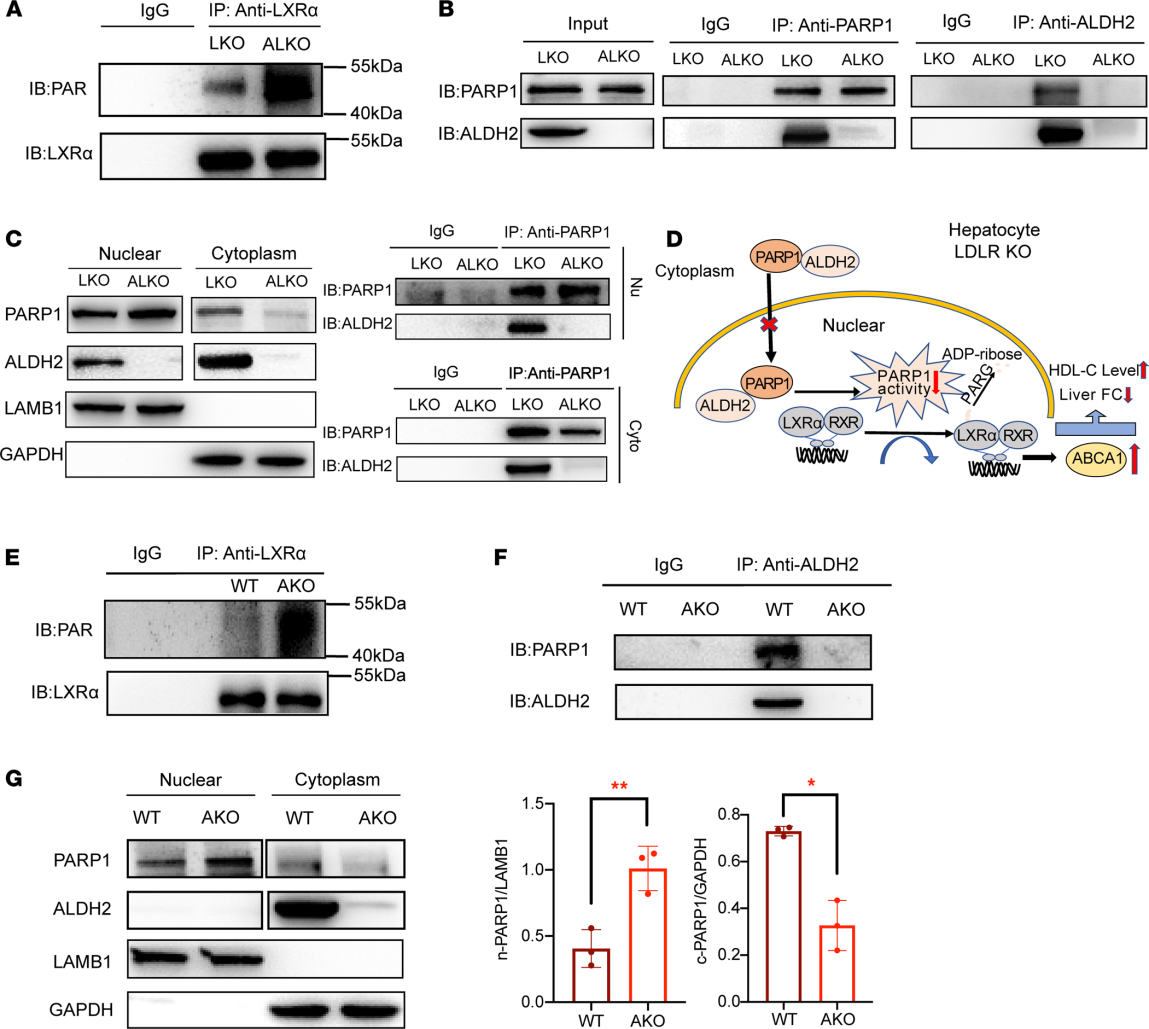
ALDH2 and PARP1 interaction modulates nuclear translocation of PARP1, affecting poly(ADP-ribosyl)ation of LXRα and ABCA1 expressions. (**A**) Poly(ADP-ribosyl)ation of LXRα in LKO and ALKO mouse liver tissue with Western diet (WD). Experiments were repeated 3 times. (**B**) IP results of ALDH2 and PARP1 in liver tissue. (**C**) ALDH2 expression affected nuclear and cytoplasmic distributions of PARP1 in the liver. (**D**) ALDH2 inhibited nuclear translocation of PARP1 through interaction with PARP1 in the LKO mouse liver. (**E**) Poly(ADP-ribosyl)ation of LXRα by PARP1 in WT and AKO liver tissue after WD. Experiments were repeated 3 times. (**F**) IP results of ALDH2 and PARP1 in cytoplasm. (**G**) ALDH2 inhibited nuclear translocation of PARP1 in WT liver tissues (WT, *n* = 3; AKO, *n* = 3). Experiments were repeated 3 times. Statistical comparisons were made using a 2-tailed Student’s *t* test or ANOVA. All data are mean ± SD. *P < 0.05, **P < 0.01. n-PARP1, nuclear PARP1; c-PARP1, cytoplasmic PARP1.

To examine whether ALDH2 deficiency decreases ABCA1 expression In LDLR-KO mice through increasing poly(ADP-ribosyl)ation of LXRα, we extracted proteins from LKO or ALKO hepatocytes and conducted IP with anti-LXRα antibody, followed by immunoblotting with an anti-PAR antibody. Indeed, poly(ADP-ribosyl)ation of LXRα was increased in ALKO liver tissue compared with LKO tissue (Figure 4A). Although we did not observe significantly different protein expression levels of PARP1 and PARG between LKO and ALKO mice (Supplemental Figure 4D and Supplemental Figure 2, A and D), PARP1 enzymatic activity was most likely responsible for poly(ADP-ribosyl)ation of LXRα.

To investigate how ALDH2 regulates poly(ADP-ribosyl)ation of LXRα by PARP1, we focused on protein interactions of ALDH2 and PARP1. We performed a proteomic study in ALDH2-enriched protein mixture and found that PARP1 was among many nuclear proteins pulled down by ALDH2 in a human liver cell line, HL-7702 (Supplemental Table 2). Consistently, we performed endogenous IP using LKO and ALKO mouse liver tissues and found that PARP1 indeed interacted with ALDH2 and PARP1 in LKO mice (Figure 4B). ALDH2 mainly exists in the mitochondria and cytoplasm (Supplemental Figure 4E); PARP1 is primarily located in the nucleus. To further examine the cellular locations of these protein interactions, we separated the nuclear and cytoplasm fractions from LKO and ALKO liver tissue and conducted IP with an anti-ALDH2 antibody followed by immunoblotting with anti-ALDH2 and anti-PARP1 antibodies, respectively. In the absence of LDLR (LKO mice), ALDH2 interacted with PARP1 in the cytoplasm and nucleus (Figure 4C). Moreover, ALDH2-KO in LDLR-KO background (ALKO) increased the nuclear proportion of PARP1 with decreased cytosolic levels, suggesting ALDH2/PARP-1 interaction attenuated nuclear translocation of PARP1. All these data suggest that ALDH2 downregulated ABCA1 expression and decreased cholesterol efflux from LKO liver tissues through attenuating poly(ADP-ribosyl)ation of LXRα, which manifested as lower levels of HDL in the circulation but increased hepatic steatosis in ALKO compared with LKO mice (Figure 4D and Figure 1, D–F).

To further support this conclusion in WT and AKO mice, we found that poly(ADP-ribosyl)ation of LXRα was increased in AKO liver tissue compared with WT tissue (Figure 4E). Consistently, immunofluorescence (IF) staining analysis of WT and AKO hepatocytes showed that ALDH2-KO (AKO) increased the nuclear PARP1 signal compared with WT hepatocytes (Supplemental Figure 4F). Accordingly, ALDH2 deficiency in AKO mice significantly increased nuclear PARP1 while PARP1 decreased in the cytoplasm (Figure 4, F and G, and Supplemental Figure 4F). In conclusion, ALDH2/PARP1 interaction attenuated nuclear translocation of PARP1, which resulted in decreased poly(ADP-ribosyl)ation of LXRα and upregulation of ABCA1; in the absence of ALDH2, increased nuclear PARP1 and LXRα poly(ADP-ribosylation) led to downregulation of hepatic ABCA1 expression and HDL biogenesis.

### PARP1 inhibition dampens hepatosteatosis and increases HDL in AKO mice.

**Figure 5:**
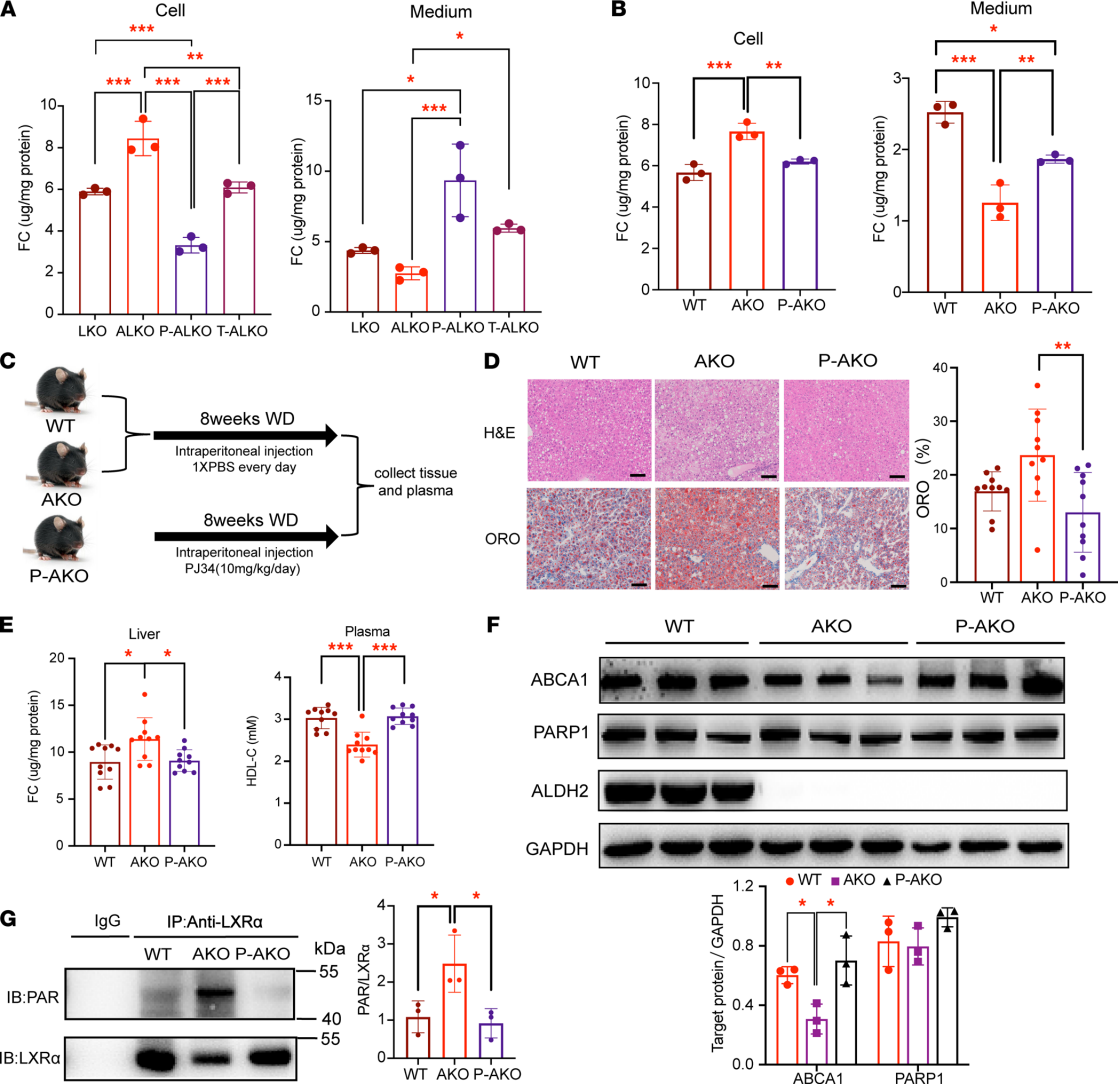
PARP1 inhibition attenuates hepatic steatosis and increases HDL-C levels in AKO mice. (**A**) Free cholesterol levels in LKO and ALKO mouse hepatocytes treated with PARP1 inhibitor PJ34 (50 μM, 24 h; P-LKO or P-ALKO) or LXRα agonist T0901317 (10 μM, 24 h, T-LKO or T-ALKO) after treating with ox-LDL (50 μg/mL, 16 h).LKO, *n* = 3; ALKO, *n* = 3; P-ALKO, *n* = 3; T-ALKO, *n* = 3. (**B**) Free cholesterol levels in WT and AKO mouse hepatocytes treated with PJ34 (50 μM, 24 h; P-AKO or P-WT) after ox-LDL treatment (50 μg/mL, 16 h). WT, *n* = 3; AKO, *n* = 3; P-AKO, *n* = 3; T-AKO, *n* = 3. (**C**) AKO or WT mice were injected with 1× PBS or PJ34 and fed with a Western diet for 8 weeks. (**D**) Representative H&E and Oil Red O staining for mouse liver tissues (WT: WT mice injected with 1× PBS; AKO: ALDH2-KO mice injected with 1× PBS; P-AKO: AKO mice injected with PJ34). WT, *n* = 10; AKO, *n* = 10; P-AKO, *n* = 10; scale bar: 100 μm. (**E**) Free cholesterol in liver tissue and HDL-C in plasma. WT, *n* = 10; AKO, *n* = 10; P-AKO, *n* = 10. (**F**) Western blotting analysis of ABCA1 and PARP1 expression in WD mouse liver tissue. WT, *n* = 3; AKO, *n* = 3; P-AKO, *n* = 3. (**G**) Poly(ADP-ribosyl)ation of LXRα in WT, AKO, and P-AKO mouse liver tissue. Experiments were repeated 3 times. Statistical comparisons were made using 1-way ANOVA followed by Student-Newman-Keuls for multiple-comparison tests. All data are mean ± SD. *P < 0.05, **P < 0.01, ***P < 0.001.

Next, we examined whether PARP1 inhibition could reverse ALDH2 deficiency–induced hepatosteatosis and decrease HDL-C levels in AKO mice with WD feeding. To do so, we first treated hepatocytes from LKO and ALKO mice with the PARP1 inhibitor PJ34 or the LXRα agonist T0901317. Consistently, LXRα activation or PARP1 inhibition significantly attenuated free cholesterol levels in cell lysates of ALKO compared with LKO mice, whereas the levels of free cholesterol secreted into the cell media were increased compared with LKO mice (Figure 5A). Similar results were observed for the PARP1 inhibition by PJ-34 treatment in AKO hepatocytes (Figure 5B). Next, we treated AKO and WT mice with PJ34 and fed them with WD for 8 weeks to test whether PARP1 inhibition could reverse ALDH2 deficiency–induced hepatosteatosis and increase HDL-C levels (Figure 5C). Interestingly, WD feeding induced similar weight gains in WT and AKO mice, and PJ34 treatment led to a significant decrease of body weight (Supplemental Figure 5, A and B). Furthermore, there was a slight increase in liver/body weight in AKO mice but no significant difference in white adipose tissues (Supplemental Figure 5, C and D). The WD-induced fatty liver due to ALDH2 deficiency was significantly prevented with PARP1 inhibition (Figure 5D). Moreover, the increased levels of free cholesterol in AKO mouse liver tissue were reversed as well as the decreased plasma HDL-C levels (Figure 5E), consistent with the fast performance liquid chromatography (FPLC) analysis of lipoprotein fractions (Supplemental Figure 5E). Interestingly, the increased triacylglycerides (TAG) levels in the VLDL fraction in AKO mice were also reversed with PJ34 treatment (Supplemental Figure 5E). As expected, the decreased expression of ABCA1 in AKO mouse liver was reversed after PJ34 treatment without significantly affecting the PARP1 expression (Figure 5F). Moreover, poly(ADP-ribosyl)ation of LXRα was increased in AKO liver tissue compared with WT tissue, which disappeared when treated with PARP1 inhibitor PJ34 (Figure 5G).

Given the importance of ApoA-I in HDL biogenesis, we next measured ApoA-I levels in cell media and lysates from the primary mouse hepatocytes and found that the levels of ApoA-I in cell lysates had no significant change, but ApoA-I levels in cell media were significantly decreased (Supplemental Figure 5F). We further detected ApoA-I in the plasma and liver tissues of WT, AKO, and P-AKO mice. Consistently, ApoA-I levels in AKO plasma were decreased compared with WT plasma, which was completely recovered with PJ34 treatment, whereas the protein levels of ApoA-I did not significantly change in the liver tissues (Supplemental Figure 5, G and H). All these data demonstrated that ALDH2 regulated ABCA1 and ApoA-I expression in HDL biogenesis, and PARP1 inhibition has the potential to raise HDL-C levels and attenuate hepatosteatosis induced by ALDH2 deficiency.

### ALDH2 attenuates the nuclear translocation of PARP1, presumably through interacting with the nuclear localization sequence of PARP1.

**Figure 6:**
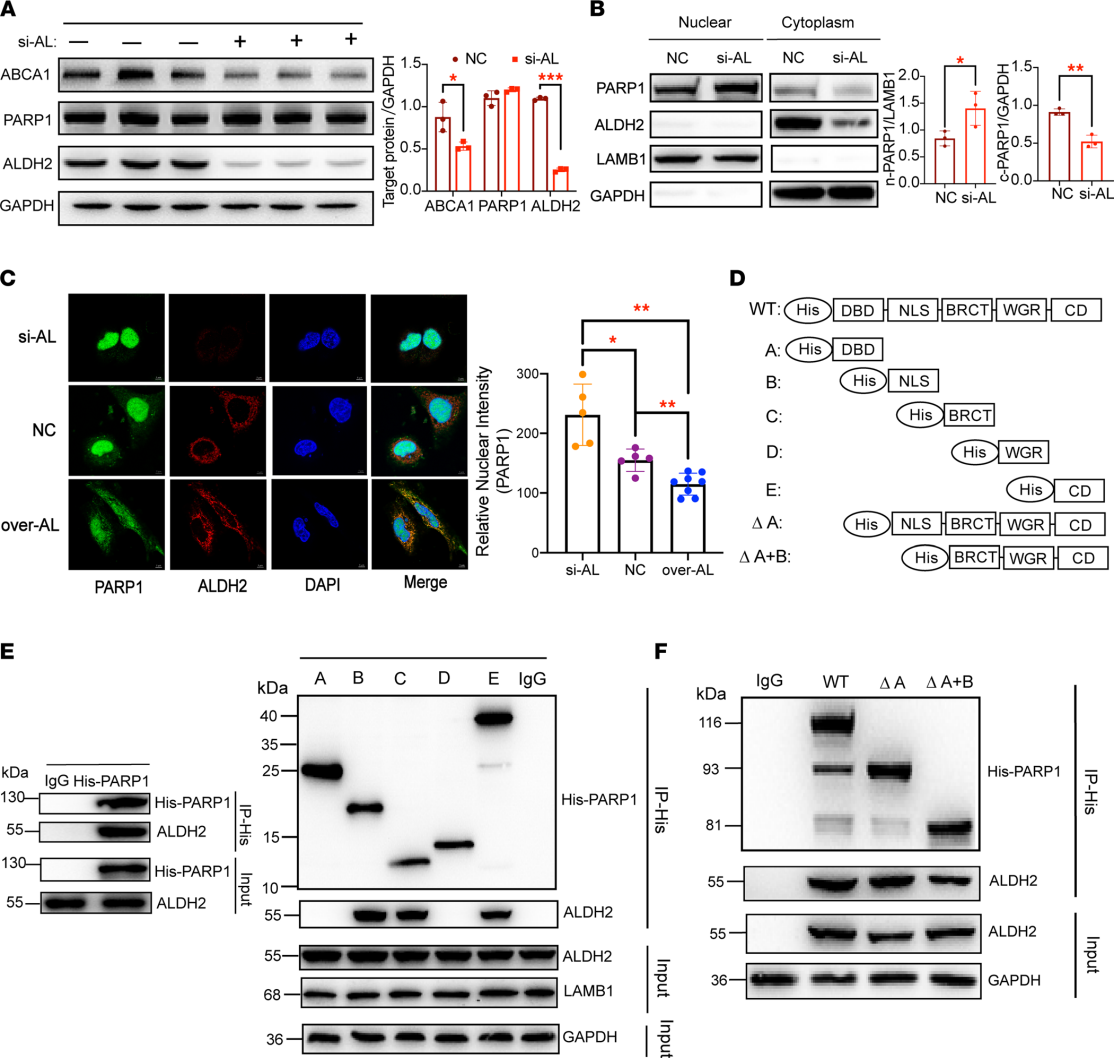
ALDH2 interacts with the nuclear localization sequence of PARP1 and attenuates nuclear translocation of PARP1. (**A**) Western blotting analysis of ABCA1 and PARP1 expression in HL-7702 cells after transfection with siNC or siALDH2 treated with ox-LDL (50 μg/mL, 16 h) (*n* = 3). (**B**) ALDH2 knockdown increased nuclear translocation of PARP1 and decreased cytosolic PARP1 (*n* = 3). Experiments were repeated 3 times. (**C**) Immunofluorescent analysis of the nuclear fraction of PARP1 in ALDH2 knockdown (si-AL, transfection with siALDH2) or ALDH2 overexpression (over-AL, transfection with Flag-ALDH2 plasmid). Red, ALDH2; green, PARP1; blue DAPI; *n* = 5; scale bar: 5 μm. (**D**) Diagram of major domains in PARP1: DBD, DNA binding domain; NLS, nuclear localization sequence; BRCT, BRCA1 C-terminus domain; WGR, arginine-rich domain; CD, catalytic domain. The schematics of the His-PARP1 expression plasmid as well as domains with truncated mutants. (**A**) 1–214 aa DBD, (**B**) 215–372 aa NLS, (**C**) 373–476 aa BRCT domain, (**D**) 525–656 aa WGR domain, (**E**) 657–1014 aa CD. (**E**) NLS, BRCT, and CD mediated the association of PARP1 with ALDH2. Pulled down ALDH2 was detected by immunoblotting. (**F**) Deletion of NLS attenuated the interactions of PARP1 with ALDH2. Experiments were repeated 3 times. Statistical comparisons were made using a 2-tailed Student’s *t* test or 1-way ANOVA followed by Student-Newman-Keuls for multiple-comparison tests. All data are mean ± SD. *P < 0.05, **P < 0.01, ***P < 0.001.

To determine the similar role of ALDH2 on ABCA1 expression in the human liver to that of mice, we transfected a human liver cell line (HL-7702) with ALDH2 siRNA to knock down the ALDH2 levels. We found that the expression of ABCA1 was significantly decreased in ALDH2 knockdown cells without significantly affecting the PARP1 expression in HL-7702 cells treated with ox-LDL (Figure 6A and Supplemental Figure 6A). Moreover, ALDH2 knockdown led to decreased levels of PARP1 in the cytosol (c-PARP1) while PARP1 increased in the nucleus (n-PARP1), suggesting that ALDH2 interacted with PARP1 in the cytoplasm and modulated nuclear translocation of PARP1 (Figure 6B). Consistently, IF staining analysis demonstrated that increased nuclear PARP1 was found in ALDH2 knockdown cells and overexpression of ALDH2 attenuated nuclear PARP1 (Figure 6C). Furthermore, more PARP1 remained in the cytoplasm in ALDH2 overexpression cells (Figure 6C and Supplemental Figure 6B). Next, we mapped the interacting sites of PARP1 that are potentially responsible for modulating the nuclear translocation of PARP1. By forced expression of His-tagged PARP1 fragments in HL-7702 cells and HEK293T cells, we mapped the ALDH2-binding sites to the DNA binding domain (1–214 aa), nuclear localization sequence (NLS) domain (215–372 aa), BRCT domain (373–476 aa), WGR domain (525–656 aa), and catalytic domain (657–1014 aa) of PARP1 as well as the truncations of the NLS and DBD domains (ΔA and ΔA+B) using IP assays (Figure 6, D and E, and Supplemental Figure 6C) (35, 36). The pulldown assay showed that His-tagged NLS (truncated form B; Figure 4D), BRCT (truncated form C), and CD (truncated form D) pulled down ALDH2 in both cell lines, indicating that ALDH2 interacted with PARP1 at multiple sites (Figure 6E and Supplemental Figure 6C). However, deletion of NLS in ΔA+B significantly attenuated the interaction with ALDH2 compared with WT or ΔA (Figure 6F). All these results suggest that ALDH2 inhibited nuclear translocation of PARP1, presumably through masking NLS of PARP1, although both proteins interacted at multiple sites.

### ALDH2 rs671 modulates HDL-C levels through increasing poly(ADP-ribosyl)ation of LXRα due to decreased interaction with PARP1.

**Figure 7:**
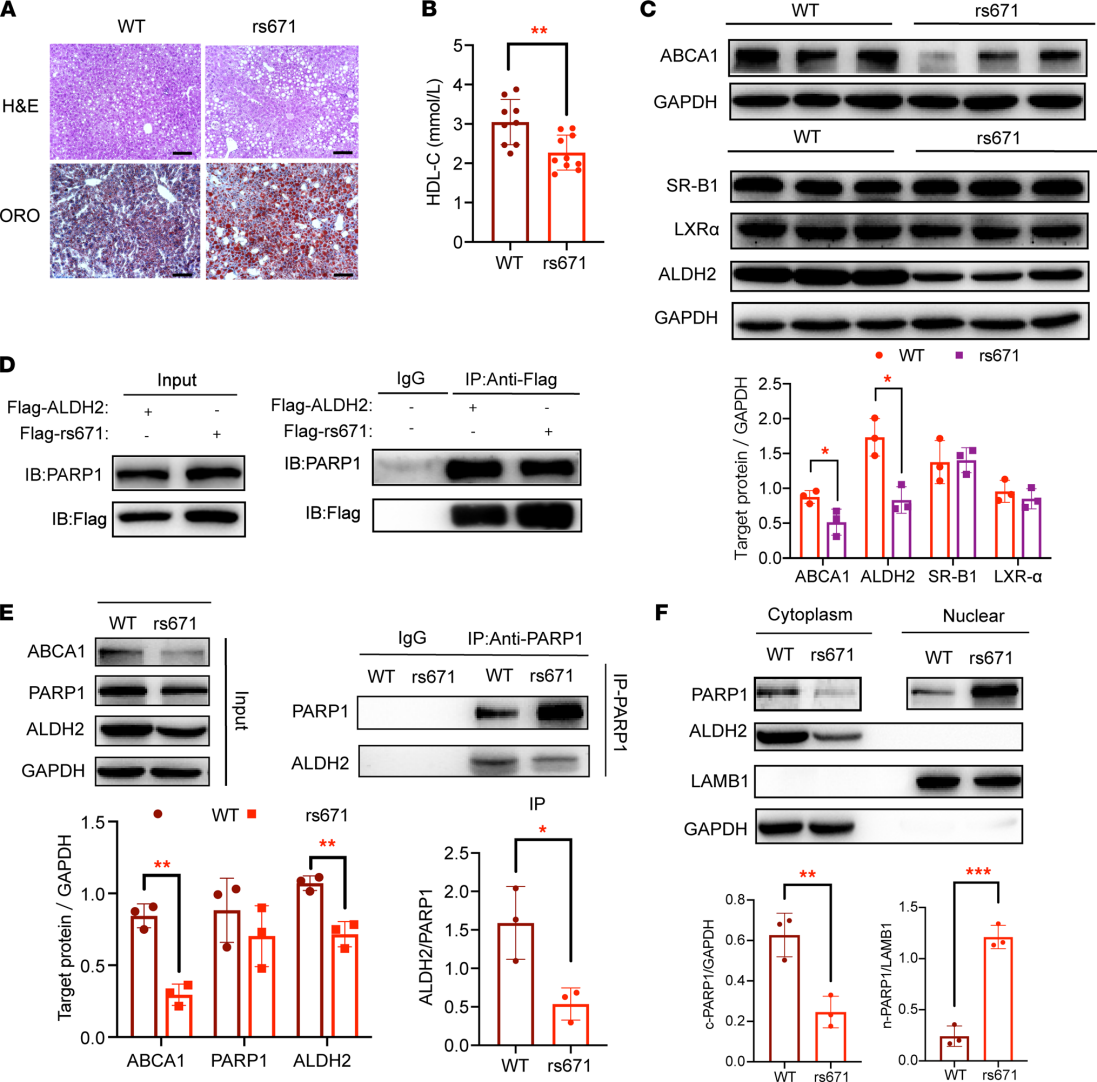
ALDH2 rs671 modulates HDL-C levels in mice and human liver through increasing poly(ADP-ribosyl)ation of LXRα due to attenuated ALDH2/PARP1 interaction. (**A**) Representative H&E and Oil Red O staining for mouse liver fed with a Western diet (WD) for 8 weeks (WT and ALDH2 rs671-KI mice, referred to as rs671). Scale bar: 100 μm. (**B**) HDL-C in plasma at 16^th^ week (WD for 8 weeks). WT, *n* = 9; rs671, *n* = 10. (**C**) Western blotting analysis of ABCA1, ALDH2, LXRα, and SR-B1 expression in mouse liver tissue. WT, *n* = 3; rs671, *n* = 3. (**D**) IP results of WT ALDH2 or ALDH2 rs671, PARP1. (**E**) IP results of ALDH2 and PARP1 in human liver tissues. WT, *n* = 3; rs671, *n* = 3. Experiments were repeated 3 times. (**F**) ALDH2 rs671 significantly increased nuclear translocation of PARP1 in human liver tissue. WT, *n* = 3; rs671, *n* = 3. Statistical comparisons were made using a 2-tailed Student’s *t* test. All data are mean ± SD. *P < 0.05, **P < 0.01, ***P < 0.001.

Next, we examined whether a similar mechanism operates in ALDH2 rs671 in regulating HDL levels. To do so, we generated ALDH2 rs671-KI mice and fed them with WD for 8 weeks. The rs671 mice gained significant weight and exhibited hepatosteatosis with lower levels of HDL-C compared with the WT mice (Figure 7, A and B, and Supplemental Figure 7A). A previous study demonstrated that mutant ALDH2 protein levels were lower in mouse liver carrying the ALDH2 rs671 SNP (Figure 7C), presumably due to faster protein turnover (37). Interestingly, ALDH2 rs671 SNP did not present in the nucleus in liver tissues (Supplemental Figure 7B), differing from the macrophages (29). However, the expression of ABCA1 was significantly decreased, whereas the protein levels of SR-B1 and LXRα had no significant change in rs671 liver tissue (Figure 7C).

Next, we investigated whether ALDH2 rs671 affects the interaction with PARP1 and found that mutated ALDH2 still interacted to a similar extent with PARP1 as WT ALDH2 in HL-7702 and HEK293T cells. As shown in Figure 7D, Flag-tagged ALDH2 or rs671 pulled down similar amounts of PARP1, while overexpression of His-tagged NLS pulled down similar amounts of Flag-tagged ALDH2 or Flag-rs671 (Supplemental Figure 7, C and D). Importantly, ALDH2 protein levels were lower in human liver tissues carrying ALDH2 rs671 SNP, and interaction between ALDH2 and PARP1 decreased in human liver tissue with ALDH2 rs671 mutants (Figure 7E). Consequently, nuclear levels of PARP1 were significantly increased, which led to a decrease in ABCA1 protein expression and HDL-C levels (Figure 7, E and F). Taken together, a similar mechanism operated in ALDH2 rs671 by which an attenuated ALDH2/PARP1 interaction, primarily due to the lower expression of mutant ALDH2, resulted in more nuclear translocation of PARP1 to promote poly(ADP-ribosyl)ation of LXRα and downregulation of ABCA1.

## Discussion

**Figure 8:**
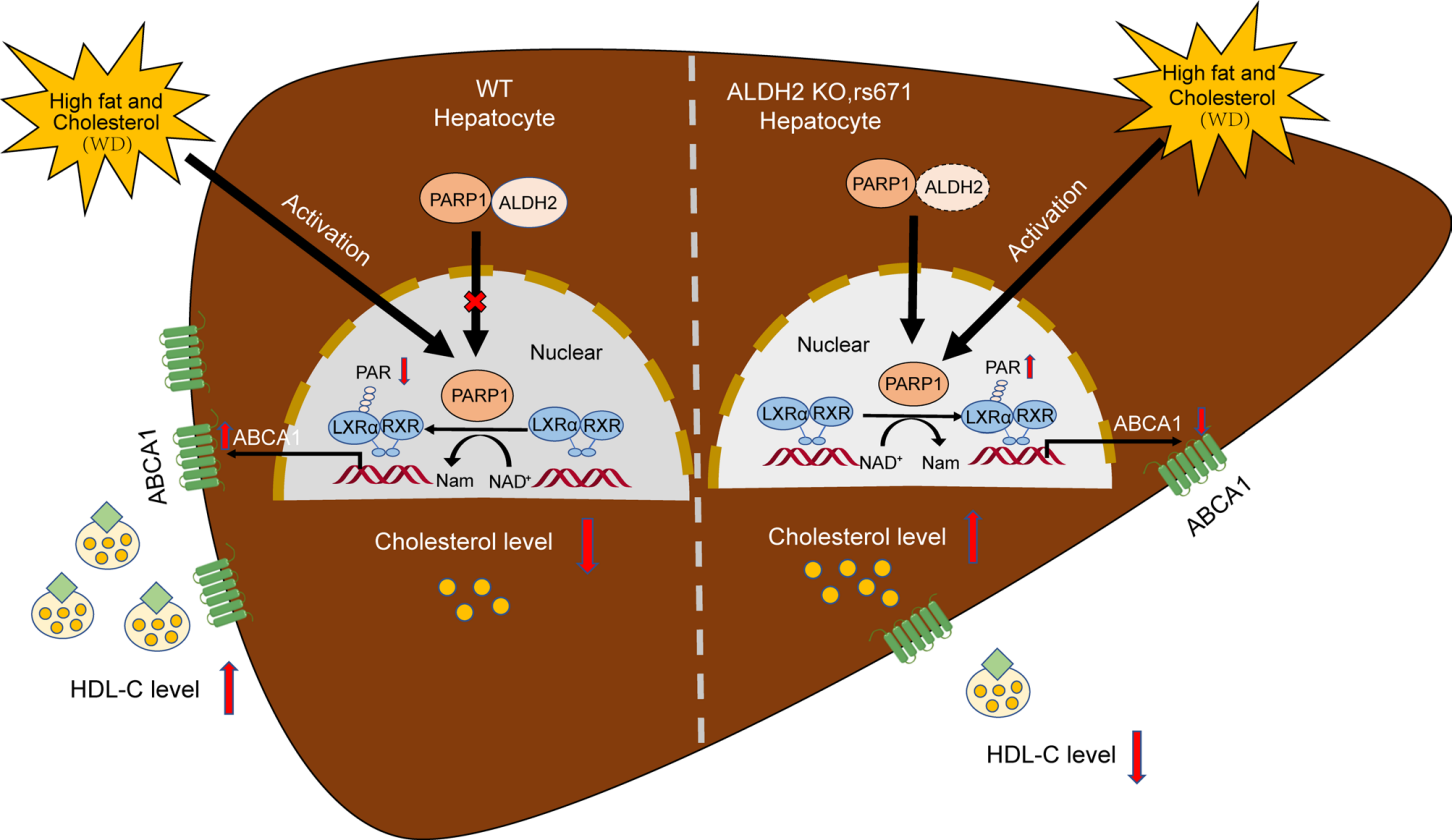
The working model of ALDH2 in modulating HDL-C levels by regulating PARP1 and LXRα-mediated ABCA1 expression with a Western diet. ALDH2 regulates hepatic HDL biogenesis and increases expression of ABCA1 through decreasing poly(ADP-ribosyl)ation of LXRα by blocking the NLS of PARP1. In ALDH2-KO or ALDH2*2 liver, attenuated ALDH2/PARP1 interaction increases nuclear translocation of PARP1 and poly(ADP-ribosyl)ation of LXRα, which leads to downregulation of ABCA1 and HDL biogenesis. This mechanism operates in the context of feeding with a Western diet that activates LXRα and PARP1.

Overwhelming experimental and clinical data support a critical role of cholesterol metabolism in the pathogenesis of atherosclerotic CVD, and cholesterol-lowering therapies remain the first-line treatment for CVD (2). Raising HDL levels has been explored as an attractive target for further lowering the residual risk for CVD; thus, understanding the mechanisms regarding HDL metabolism is critical for developing effective therapy (38, 39). Furthermore, accumulating human data have linked increased CVD risks in individuals carrying ALDH2 SNP rs671, but the underlying molecular mechanisms beyond alcohol consumption remain poorly defined (29, 33). In this study, we identified a mechanism by which ALDH2 regulates hepatic HDL biogenesis via the LXRα/PARP1/ABCA1 pathway: ALDH2 interaction with PARP1 attenuated the nuclear translocation of PARP1 to modulate LXRα activity and ABCA1 expression through poly(ADP-ribosyl)lation of LXRα; ALDH2 deficiency or mutation attenuated this interaction, which led to higher levels of PARP1 in the nucleus and downregulation of ABCA1 and HDL biogenesis (Figure 8). A similar mechanism appears to operate in human liver tissues with ALDH2 rs671. Importantly, pharmacological inhibition of PARP1 by PJ34 elevated HDL-C levels in AKO mice and attenuated hepatosteatosis after WD feeding. This study has uncovered ALDH2 as a potentially novel factor in HDL biogenesis, which provides a mechanistic link between the decreased HDL-C levels in individuals carrying ALDH2 rs671.

Epidemiological data have linked circulating HLD-C levels to the risks of CVD, but gaining genetic insights into the complex HDL biology remains a tremendous challenge (12, 40). Genetic polymorphisms of human ALDH2 have been well surveyed among a wide range of ethnic groups. Around 35%–45% of Asians and 8% of the world population carry the rs671 mutation, which is responsible for alcohol flushing syndrome due to significantly reduced enzyme activity to metabolize acetaldehyde after alcohol consumption (24). Clinical research has found that an increased risk of CVD is positively correlated with the ALDH2 rs671 SNP, especially in East Asian populations (41–43). The underlying molecular mechanisms linking ALDH2 to CVD remain poorly defined, although previous studies have focused on the aldehyde toxicity in humans carrying ALDH2 rs671 (25, 27). Recent studies identified diverse mechanisms that may be responsible for the increased CVD risks beyond alcohol consumption, including the interaction of ALDH2 with LDLR and AMPK to regulate macrophage foam cell formation (29). Interestingly, an exosome chip meta-analysis identified that the ALDH2 rs671 SNP is among the East Asian–specific coding variants that contribute to lipid levels and CVD (30, 44). Furthermore, ALDH2*2 allele mutations appear to be associated with low HDL-C levels and high levels of LDL-C (31). Our recent study found that ALDH2 regulates de novo cholesterol synthesis by affecting the stability of HMGCR, the rate-limiting enzyme in cholesterol synthesis and the target of statins (33). However, it remains to be explored how ALDH2 is involved in HDL metabolism.

In this study, we discovered that ALDH2 is an important factor in regulating HDL biogenesis in the liver through the LXRα/PARP1/ABCA1 axis, especially when LXRα and PARP1 are activated with WD feeding. Although the exact extent by which this mechanism affects HDL biogenesis in humans remains to be determined in clinical studies, our animal experimental data demonstrated an approximate 30%–40% reduction in HDL-C levels in AKO, ALKO, and rs671-KI mice after WD feeding for 26 weeks (Figure 1, A and D, and Figure 7B). Previous studies demonstrated that hepatic ABCA1, but not macrophage or other hematopoietic cells, plays a key role in regulating HDL biogenesis and circulating HDL levels (14). Liver-specific KO of ABCA1 led to an 80% decrease of the HDL-C level, and specific inhibition of liver ABCA1 enzyme activity resulted in a 40% drop of plasma HDL-C (45, 46). However, knocking out ABCA1 in the intestine decreased mouse plasma HDL-C by only 30% (47). On the other hand, transplanting WT mouse bone marrow into ABCA1-KO mice did not significantly alter plasma HDL-C levels (48). It is noteworthy that ABCA1 downregulation in ALDH2-deficient mice may not directly contribute to hepatosteatosis. Instead, stabilization of HMGCR in ALDH2-KO or rs671-KI mice is likely the major driver for hepatic lipid accumulation (33). Consistently, a previous study found that liver-specific ABCA1-KO mice were protected from hepatosteatosis (49). Although the nuclear receptor LXRα has been shown to play a critical role in the transcriptional control of lipid metabolism, especially in cholesterol homeostasis (17), it remains poorly defined how LXRα regulates HDL biogenesis through ABCA1 in the context of CVD. In addition to responding to oxysterol, our previous study found that cholesterol ester hydroperoxides, a major component in oxidized LDL, increases the circulating LDL-C through inhibiting cholesterol uptake in macrophages and hepatocytes via LXRα/LDLR pathways (50). As a major DNA repair enzyme, PARP1 has been shown to play an important role in lipid metabolism and related diseases (21). Emerging evidence points to a delicate role of PARP1 in the posttranslational modification of LXRα in cholesterol metabolism. On the one hand, PARP1 represses LXRα-mediated ABCA1 expression and cholesterol efflux in macrophages (22). On the other hand, PARP1 activation suppresses LXRα and prevents upregulation of genes associated with high-cholesterol diet–induced cholesterol disposal, whereas inactivated PARP1 is indispensable for LXRα transactivation (23). In this study, we found that ALDH2 is a critical factor in regulating nuclear translocation of PARP1 and subsequent poly(ADP-ribosyl)ation of LXRα, which leads to ABCA1 expression and HDL biogenesis (Figure 8). These effects are achieved through a direct protein-protein interaction between ALDH2 and PARP1 (Figure 6). Although ALDH2 interacts with multiple sites in PARP1, the association of ALDH2 with the NLS of PARP1 appears consequential for the nuclear translocations of PARP1. Interestingly, in the mouse liver of ALDH2 rs671 or human liver, ABCA1 was downregulated because of the increased nuclear presence of ALDH2 primarily owing to the lower expression of ALDH2 proteins of the rs671 mutant, and the interaction of PARP1 and mutant ALDH2 was not significantly affected (Figure 7). Understanding the factors regulating HDL metabolism will likely have profound clinical impact on developing therapeutic strategies aimed at raising HDL. A low level of HDL cholesterol is an important predictor of atherosclerotic cardiovascular events, and the National Cholesterol Education Program defines HDL level less than 40 mg/dL as a clear risk factor for CVD (51). Despite aggressively lowering cholesterol levels by statins, there are still some residual risk factors in CVD (52). Thus, raising HDL-C has been explored therapeutically. Unfortunately, most efforts to raise HDL-C, including fibrates, niacin, and cholesteryl ester transfer protein inhibitors, have failed to provide expected clinical benefits in CVD outcomes (53). Although the underlying mechanisms for these unsatisfied clinical trials remain to be clearly defined, it appears challenging to therapeutically improve HDL functions, such as reverse cholesterol transport (RCT), antioxidant, and antiinflammation, in addition to elevating HDL-C levels (13, 14). In this study, we found that ALDH2 deficiency in AKO, ALKO, or rs671-KI mice led to decreased HDL-C when fed with WD primarily through downregulation of ABCA1 as a result of increased nuclear translocation of PARP1 and poly(ADP-ribosyl)ation of LXRα. Excitingly, pharmacological inhibition of PARP1 by the small molecular compound PJ34 successfully increased HDL-C levels in AKO mice by restoration of ABCA1 and ApoA-I expression, 2 key players in HDL biogenesis. Notably, the PARP inhibitor olaparib has recently been approved by the FDA in the United States and the European Medicines Agency (EMA) as a maintenance therapy for patients with germline BRCA1 or BRCA2 mutations in metastatic pancreatic cancer (54). Conceivably, these compounds have the potential to be tailored to raise HDL-C in the context of CVD. Furthermore, ALDH2 activator has been actively explored for prevention and treatment of CVD, especially for individuals carrying SNP rs671. To examine whether ALDH2 enzymatic activity affects the protein expression of ALDH2 and/or interactions with PARP1 to influence HDL-C levels through the LXRα/PAPR1/ABCA1 axis, we used ALDH2 inhibitor disulfiram or activator Alda1 to treat human liver cell line HL-7702 and found that neither treatment altered ABCA1 and ALDH2 protein levels (Supplemental Figure 7E), suggesting that ALDH2 enzymatic activity plays a limited role in ALDH2 and ABCA1 expression in vitro. However, it remains to be studied in clinical settings whether ALDH2 activation improves HDL-C levels as well as CVD outcomes.

In summary, our research revealed a mechanism by which ALDH2 interaction with PARP1 regulates ABCA1 expression and hepatic HDL biogenesis through decreasing poly(ADP-ribosyl)ation of LXRα by blocking NLS of PARP1. Importantly, this study provides a mechanistic link for the reverse association of HDL-C in individuals carrying ALDH2 rs671 mutation. In addition, targeting the LXRα/PARP1/ABCA1 axis by PARP1 inhibitors successfully raised HDL-C in the plasma and attenuated hepatosteatosis in AKO mice after WD feeding.

## Methods

The details of experimental methods, including histological analysis, cell culture, real-time PCR (Supplemental Table 1), Western blotting, IP, immunofluorescence microscopy, FPLC, and metabolomics, can be found in the supplemental material.

### Antibodies.

Antibodies against ALDH2 (15310-1-AP), LDLR (10785-1-AP), LXRα (14351-1-AP), GAPDH (60004-1-Ig), DYKDDDDK Tag (80010-1-RR), ABCG5 (27722-1-AP), ApoA-I (66206-1-Ig), His-Tag (66005-1-1g), lamin B1 (12987-1-AP), and α-tubulin (11224-1-AP) were purchased from Proteintech. Antibodies against poly/mono-ADP ribose (83732S), PARP1 (46D11), and ABCA1 (E7X5G) were purchased from Cell Signaling Technology. LXRα/β (sc-377260) and PARG (sc-398563) were purchased from Santa Cruz Biotechnology. SR-B1 (NB400-104SS) was purchased from Novus Biologicals. Antibodies against CYP7A1 (ab65596) were purchased from Abcam.

### Animals.

All animal experiments were approved by the IACUC of SINH, CAS. All mice were housed in a temperature-controlled and pathogen-free room under a 12-hour light/12-hour dark cycle. Mice used in the present study were on a C57BL/6J background. ALDH2-KO mice were a gift from Jun Ren and Aijun Sun at Zhongshan Hospital (affiliated with Fudan University, Shanghai, China), and rs671-KI mice were a gift from Yong Cang at ShanghaiTech University, Shanghai, China. Genome-edited F0 ALDH2^–/–^ mice were backcrossed with C57BL/6J mice for 2 generations. WT and ALDH2^–/–^mice were obtained by breeding ALDH2^+/–^. ALDH2^–/–^ LDLR^–/–^ mice were obtained by crossing ALDH2^–/–^ mice and LDLR^–/–^ mice. WT and ALDH2*2 mice were obtained by breeding ALDH2*1/*2 mice. Six-week-old male, WT, AKO (ALDH2^–/–^), LKO (LDLR^–/–^), and ALKO (ALDH2^–/–^ LDLR^–/–^) mice were fed with WD (Research Diets, D12079B) or CD for 26 weeks, while 8-week-old male WT and rs671 (ALDH2*2) mice were fed with WD for 8 weeks. For PJ34 experiments, 7-week-old male WT and AKO mice were fed with WD and administered PJ34 (10 mg/kg/day) or vehicle (normal saline) by peritoneal injection once a day for 8 weeks.

### Human study.

Human samples were from a previous study (55), and we used normal liver tissues from patients with liver cancer undergoing liver surgery. Samples were genotyped for the variant-rs671 through use of custom Illumina Golden Gate or Affymetrix Axiom arrays at BGI Inc.

### Histological analyses.

Liver tissues were fixed in 4% formaldehyde. After dehydration, liver tissues were either embedded in paraffin or embedded in OCT (Tissue-Tek, Sakura Finetek) and then frozen in dry ice. The sections (5 μm) of liver tissue were subjected to H&E staining. For Oil Red O staining, the sections (10 μm) of liver tissue were stained with Oil Red O (O0625, Sigma-Aldrich) and counterstained with Mayer’s hematoxylin to visualize intracellular lipid droplets. All images were obtained with a light microscope (Vectra2, PerkinElmer) and analyzed with ImageJ (NIH) for quantitative measurements.

### Cell culture.

Primary hepatocytes were isolated from mice, purified, and cultured as previously described (56). Briefly, mice (CD) were anesthetized by i.p. injection of 6% chloral hydrate (10 μL/g) and perfusion buffer and enzyme buffer (collagenase type 1, 0.3 mg/mL, Worthington Biochemical). Finally, we used Percoll (GE Healthcare) to isolate mouse hepatocytes and cultured these hepatocytes in DMEM with 10% FBS and 1% penicillin-streptomycin solution (PS). All hepatocytes or 7702 cells for detecting expression of ABCA1 and free cholesterol were pretreated with 50 μg/mL ox-LDL for 16 hours. Cell lines 7702 and HEK293T were purchased from the CAS Cell Bank and cultured in DMEM containing 10% FBS.

### Real-time PCR.

Total RNA was isolated from liver tissue using TRIzol reagent (9109, Takara) and organic reagent. The purity of extracted total RNA was determined by the A260/A280 ratio, and 1 mg of purified RNA from each sample was transcribed to cDNA. Gene expression was normalized to β-actin expression and calculated using the comparative CT method. The primer sequences are listed in Supplemental Table 1.

### IP experiment.

Human liver cell line 7702 or HEK293T cells were transfected with target tagged protein using Attractene Transfection reagent (301005, Qiagen), incubated by IP cell lysis buffer (50 mM Tris-HCL, 400 mM NaCl, 0.8% Triton X-100, pH 7.5), and cleared with His-beads (B23601, Biotool) overnight. For hepatocytes and liver tissue, target protein was precipitated with respective antibodies and protein A/G beads (B23202, Biotool). Cytoplasmic and nuclear proteins were acquired by using the Nuclear and Cytoplasmic Protein Extraction kit (P0027, Beyotime) according to the manufacturer’s instructions.

### IF microscopy.

Cells were fixed with 10% formaldehyde, pretreated with 0.5% Triton X-100, blocked with 1% BSA, and incubated with primary antibodies recognizing ALDH2 (1:100) and PARP1 (1:100). Secondary antibodies were used to detect primary antibody–antigen complexes with different color combinations as needed. Finally, cover glass was mounted on slides using ProLong Diamond Antifade Mountant (Molecular Probes, P36970). Images were acquired using Zeiss LSM 780.

### Total cholesterol, HDL, V-LDL, ApoA-I, and LDL-C assays.

Plasma of total cholesterol, HDL-C, ApoA-I, and LDL-C was detected total cholesterol assay kit, high-density lipoprotein cholesterol assay kit, Apolipoprotein A-I Assay Kit, and low-density lipoprotein cholesterol assay kit (Nanjing Jiancheng Bioengineering Institute). The free cholesterol and total cholesterol in liver and cells were detected by gas chromatography–mass spectrometry (GC-MS). Liver tissues (10 mg–20 mg) and hepatocytes (1 × 10^6^) were homogenized in 1× PBS. Cholesterol was extracted at m/z = 329, 368, 458, and Internal standard (IS) was extracted at m/z = 217, 357 by GC chromatograms.

### FPLC.

The combined plasma samples of 8 mice in each group were separated by FPLC using a sucrose 6 10/300 GL column (GE Healthcare). Forty fractions (0.5 mL each) were collected. Triglyceride and cholesterol concentrations were measured in each fraction. Fractions 19 through 24 contained VLDL, fractions 25 through 30 contained IDL/LDL, and fractions 31 through 40 contained HDL.

### The extraction of total and free cholesterol for GC-MS analysis.

Total and free cholesterol were analyzed by GC-MS according to previously published protocols from our lab (33).

### Nontarget metabolomics.

Sample preparation, data acquisition, and analysis were performed according to a previously published protocol from our laboratory (34).

### Statistics.

Data are expressed as mean ± SD or mean ± SEM. Statistical analysis was conducted using 1-way ANOVA followed by Student-Newman-Keuls for multiple-comparison tests or an unpaired, 2-tailed Student’s *t* test by using Graph Pad Prism 7.0. Significant differences were considered to be *P* less than 0.05.

### Study approval.

All animal experimental procedures conformed to the NIH *Guide for the Care and Use of Laboratory Animals* (National Academic Press, 2011) and were approved by the IACUC of the Shanghai Institutes for Biological Sciences of CAS (approval 2015-AN-2). All human samples were from a previous study (55); written informed consent was obtained.

## Author contributions

HY designed this study. LL, RL, SZ, NL, LZ, XS, XX, XC, SC, and YT performed experiments. LL and HY wrote the paper.

## Supplementary Material

Supplemental data

## Version 1 04/08/2022

Electronic publication
