# Supplementary material for: Aldehyde dehydrogenase 2 and PARP1 interaction modulates hepatic HDL biogenesis by LXRα-mediated ABCA1 expression
Source: JCI Insight. 2022 Apr 8;7(7):e155869. doi: 10.1172/jci.insight.155869 (PMC9057588; doi:10.1172/jci.insight.155869)
Supplement: Supplemental data [file jciinsight-7-155869-s120.pdf]

Supplemental Tables and Figures

Supplemental Table 1. Primer sequences for mice

mouse	Forward(5'-3')	Reverse(5'-3')
ALDH2-Geno	CGGGAATTGAACTTGGTAGCCAG	GCGTAAGGCATGCGCCATCAC
ALDH2-Seq	CCTGAGCCGAATGCTTTTAAG	CTCACGCTCCTTACTGGAC
SREBP2	TGGGGACAGATGCCAAGA TG	CACCAGACTGCCCAAGTCGA
ABCA1	GCTTGTTGGCCTCAGTTAAGG	GTAGCTCAGGCGTACAGAGAT
ABCA9	TGGCTATCTTCATCCTTACCGTCT	ATGTCAAACCCCAGACCTCTCAA
SR-B1	GGAGCATTCCTTGTTCCTAGAC	CCGATGCCCTTGACAGATTT
HMGCR	AGCTTGCCCGAATTGTATGTG	TCTGTTGTGAACCATGTGACTTC
HMGCS	AGGACATCAACTCCCTGTGC	TCAGTGTTGCCTGAATCCTG
ABCG5	AGAGGGCCTCACATCAACAGA	CTGACGCTGTAGGACACATGC
ABCG8	GTCCAACACTCTGGAGGTCAG	TAGATTTCGGATGCCCAGCTC
CYP7A1	GCTGTGGTAGTGAGCTGTTG	GTTGTCCAAAGGAGGTTCACC

Supplemental Table 2 Nuclear proteins identified in ALDH2 enriched protein complex from HL7702 cell

Location	protein			
Nuclear	Negative elongation factor E	RNA helicase	Lariat debranching enzyme	Mitochondrial transcription factor A
	Mortality factor 4-like protein 1	Probable 28S rRNA (cytosine(4447)-C(5))-methyltransferase	RNA-binding protein 7	Myocyte enhancer factor 2D/deleted in azoospermia associated protein 1 fusion protein
	Zinc finger CCCH domain-containing protein 8	NF-kappa-B-repressing factor	Scaffold attachment factor B1	NCOR1 protein
	Transcriptional repressor p66-beta	Nucleolin, isoform CRA_b	Transcription factor SOX-6	Nucleosome assembly protein 1-like 1
	Transformer-2 protein homolog beta	Cold inducible RNA binding protein	Zinc finger protein 423	Poly [ADP-ribose] polymerase
	PHD finger protein 5A, isoform CRA_a	EBNA1 binding protein 2, isoform CRA_d	Heterogeneous nuclear ribonucleoprotein A0	Polypyrimidine tract-binding protein 1
	cGMP-dependent protein kinase	Leydig cell tumor 10 kDa protein homolog	Heterogeneous nuclear ribonucleoprotein H3	Protein lyl-1
	Putative nuclear envelope pore membrane protein POM 121B	PC4 protein	RNA-binding protein 5	Protein Red
	ATP-dependent RNA helicase DDX42	Ribosome biogenesis protein BRX1 homolog	tRNA (adenine(58)-N(1))-methyltransferase non-catalytic subunit TRM6	RAN binding protein 17, isoform CRA_b
	Serine/arginine repetitive matrix protein 1	WD repeat-containing protein 74	40S ribosomal protein S19	RNA-binding protein 6
	Spliceosome RNA helicase DDX39B	pre-rRNA processing protein FTSJ3	Non-POU domain containing octamer-binding isoform 1	RNA-binding region (RNP1, RRM) containing 2, isoform CRA_b
	THO complex subunit 4	NHP2-like protein 1	Splicing factor 45	Round spermatid basic protein 1-like protein
	Luc7-like protein 3	Chromodomain-helicase-DNA-binding protein 6	Histone cluster 1, H1e	Small ubiquitin-related modifier 4
	RNA-binding protein 10	DNA-binding protein SATB	Histone H2A	Uncharacterized protein
	Putative RNA-binding protein Luc7-like 2	Far upstream element-binding protein 2	5'-3' exoribonuclease	Uncharacterized protein PAPOLG
	Processing of 1, ribonuclease P/MRP subunit (S. cerevisiae)	Heterogeneous nuclear ribonucleoprotein U-like protein 1	AT rich interactive domain 4B (RBP1-like), isoform CRA_a	Zinc finger protein 229
	ATP-dependent RNA helicase DHX33	Splicing factor 1, isoform CRA_h	Bcl-2-associated transcription factor 1	Zinc finger protein 445
	Chromosome 12 open reading frame 31, isoform CRA_a	Transformer-2 protein homolog beta	cDNA FLJ51295, highly similar to Cell division cycle 5-like protein	Zinc finger protein 526
	Lysine-rich nucleolar protein 1	U2 snRNP-associated SURP motif-containing protein	cDNA FLJ56545, highly similar to ATP-dependent RNA helicase DDX50	Zinc finger protein 782
	Nucleolar GTP-binding protein 2	Cleavage stimulation factor subunit 3	HNRNPL protein	WD repeat-containing protein 70
	p21-activated protein kinase-interacting protein 1	KH domain-containing, RNA-binding, signal transduction-associated protein 3	Ligand-dependent nuclear receptor corepressor-like protein	cDNA FLJ44920 fis, clone BRAMY3011501, highly similar to Heterogeneous nuclear ribonucleoprotein U

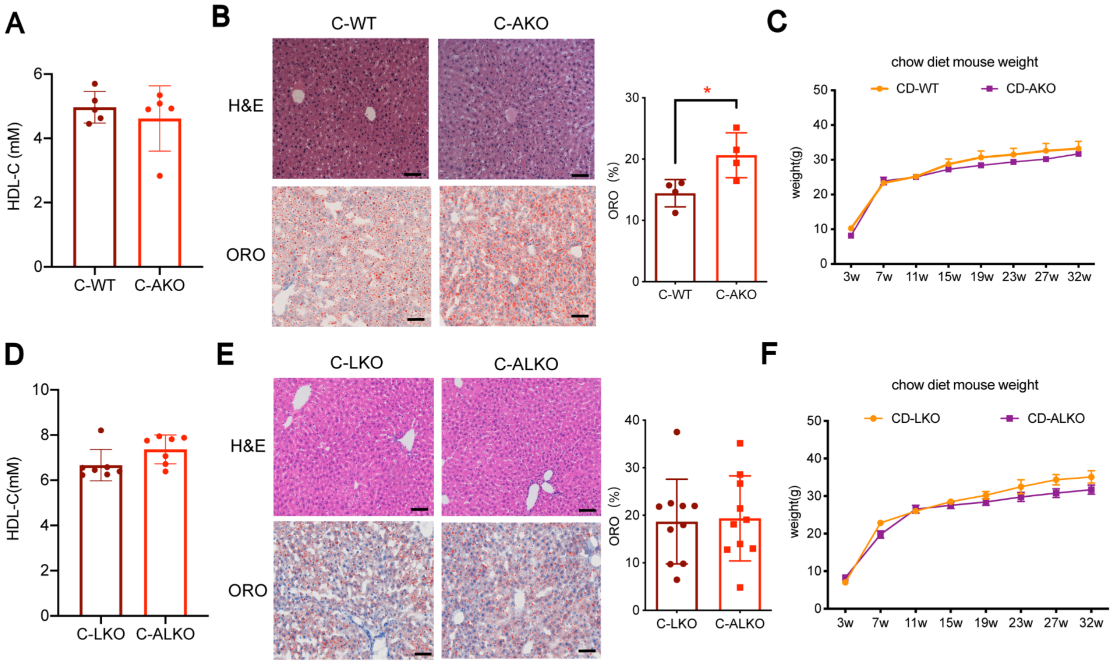

Supplemental Figure 1. ALDH2-KO does not have significant effects on HDL-C levels in LDLR-KO background or wild-type mice (WT) when fed with Chow Diet for 26 weeks. (**A**) HDL-C in WT and AKO mouse plasma at 32th (Chow Diet for 26 weeks). (C-WT, n=5; C-AKO, n=5). (**B**) Representative H&E and Oil Red O staining for WT and AKO mouse liver. (C-WT, n=4; C-AKO, n=4). (**C**) Body weights in WT and AKO mice. (**D**) HDL-C in LKO and ALKO mouse plasma at 32th. (C-LKO, n=6; C-ALKO, n=6). (**E**) Representative H&E and Oil Red O staining for LKO and ALKO mice liver (Chow Diet 26 weeks, C-LKO, n=10; C-ALKO, n=10). (**F**) Body weights LKO and ALKO mice (Chow Diet for 26 weeks). Statistical comparisons were made using a 2-tailed Student's t test. All data are mean ± SD. *P < 0.05, **P < 0.01, ***P < 0.001. Abbreviations: C-WT, wild type mice with Chow Diet; AKO, ALDH2 knockout; LKO, LDLR KO; ALKO: ALDH2/LDLR KO; H & E: Hematoxylin and Eosin.

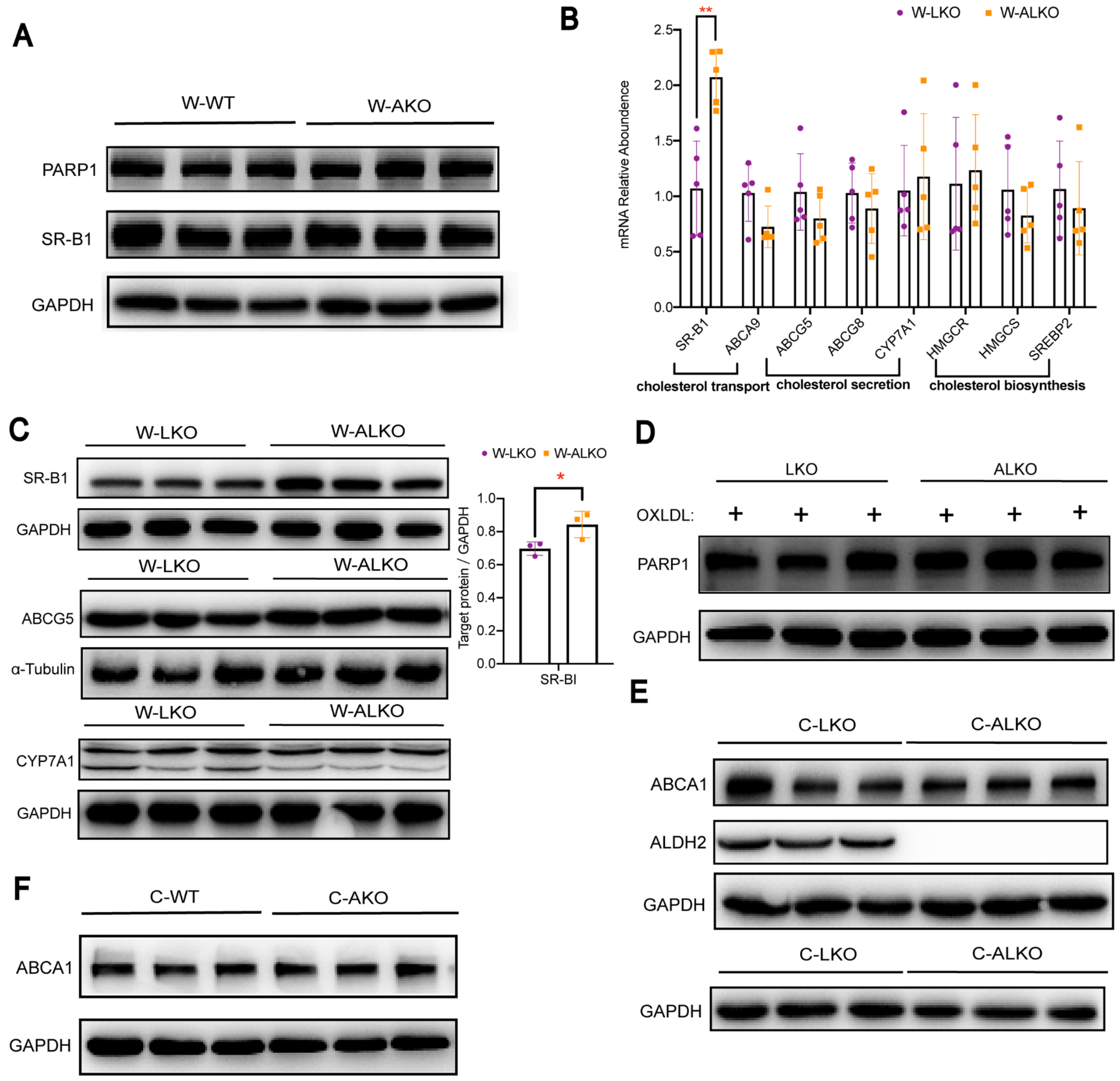

Supplemental Figure 2. Protein and RNA expressions of key proteins in cholesterol metabolism in ALDH2 and LDLR genetic knockout mice. (**A**) Western blotting analysis of PARP1 and SR-B1 expression in WT and AKO liver tissue at 32nd week (Western Diet for 26 weeks, n=3). (**B**) Hepatic mRNA levels of genes in LKO and ALKO mice at 32nd week (Western Diet for 26 weeks, n=5). (**C**) Western blotting analysis of SR-B1, ABCG5 and CYP7A1 expressions in LKO and ALKO liver tissue. (n=3). (**D**) Western blotting analysis of PARP1 expression in LKO and ALKO hepatocytes treated with ox-LDL (50 µg/ml, 16 h, n=3). (**E**) Western blotting analysis of ABCA1 expression in LKO and ALKO liver tissue at 32nd week (Chow Diet for 26 weeks, n=3). (**F**) Western blotting analysis of ABCA1 expression in WT and AKO liver tissue at 32nd week (Chow Diet for 26 weeks, n=3). Statistical comparisons were made using a 2-tailed Student's t test. All data are mean ± SD. *P < 0.05, **P < 0.01, ***P < 0.001.

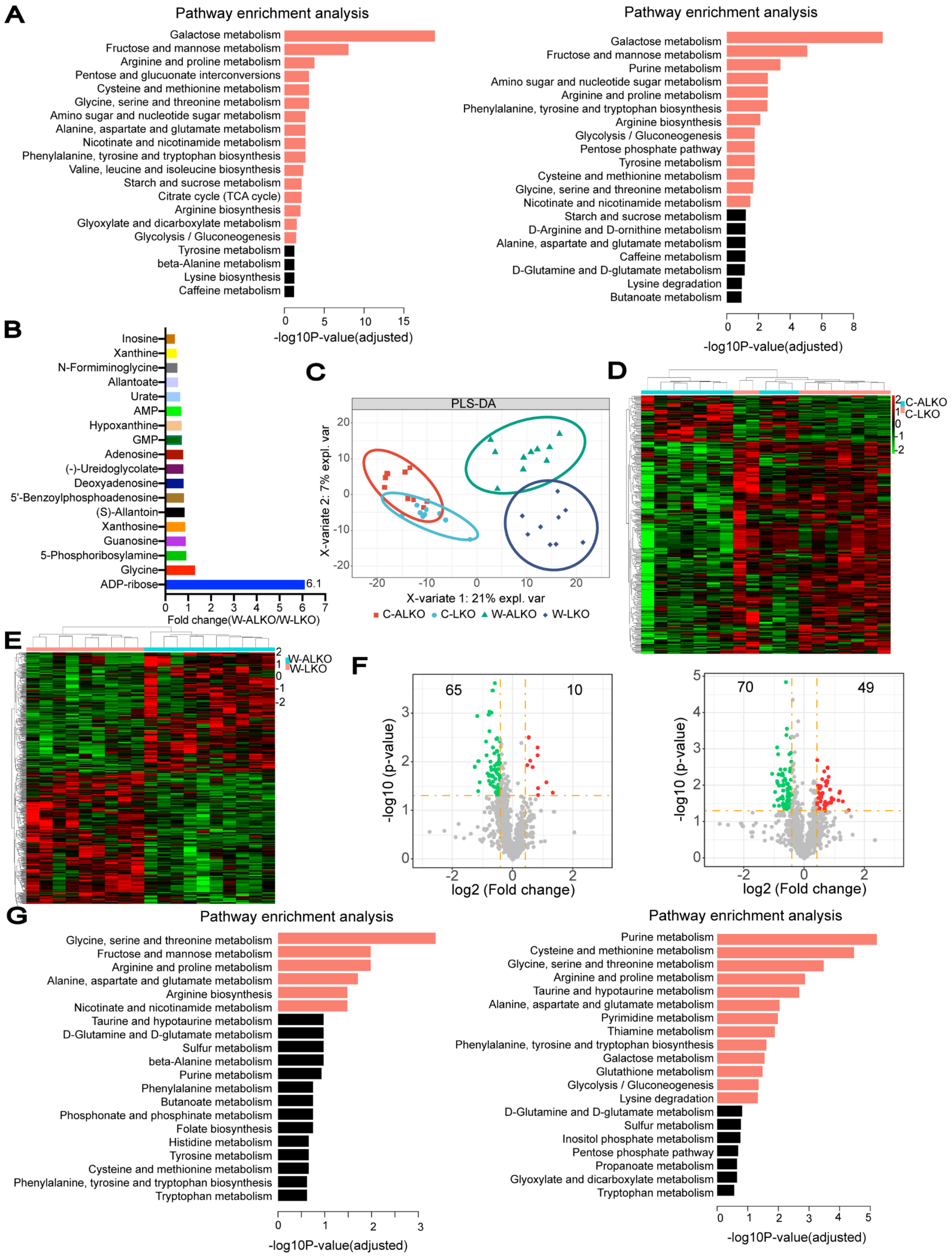

Supplemental Figure 3. Untargeted metabolomics analysis of LKO and ALKO plasma and liver tissues. (**A**) The pathway enrichments in LKO and ALKO liver tissue at 32nd weeks with Chow diet and Western Diet, respectively. The pathways shown in orange with statistical significance adjusted for false discovery rate (FDR=0.05). (**B**) List of significantly changed metabolites in purine pathway in LKO and ALKO liver tissue at 32nd week (Western Diet for 26 weeks). (**C**) PLS-DA: Partial least squares

discrimination analysis. (**D-E**) The heatmap presentation of differential metabolites in LKO and ALKO plasma at 32nd weeks with Chow diet and Western Diet. (**F**) The volcano plot of differential metabolites in LKO and ALKO plasma at 32nd week (Chow diet for 26 weeks). Red dots: upregulated metabolites ($p<0.05$); green dots: down-regulated metabolites ($p<0.05$). The volcano plot of differential metabolites in LKO and ALKO plasma at 32nd week (Western Diet for 26 weeks). The horizontal dotted line represents FDR=0.05; the vertical dotted lines represent fold changes with 3/4 or 4/3, respectively. (**G**) The pathway enrichment in LKO and ALKO plasma at 32nd week (Chow diet and Western Diet 26 weeks).

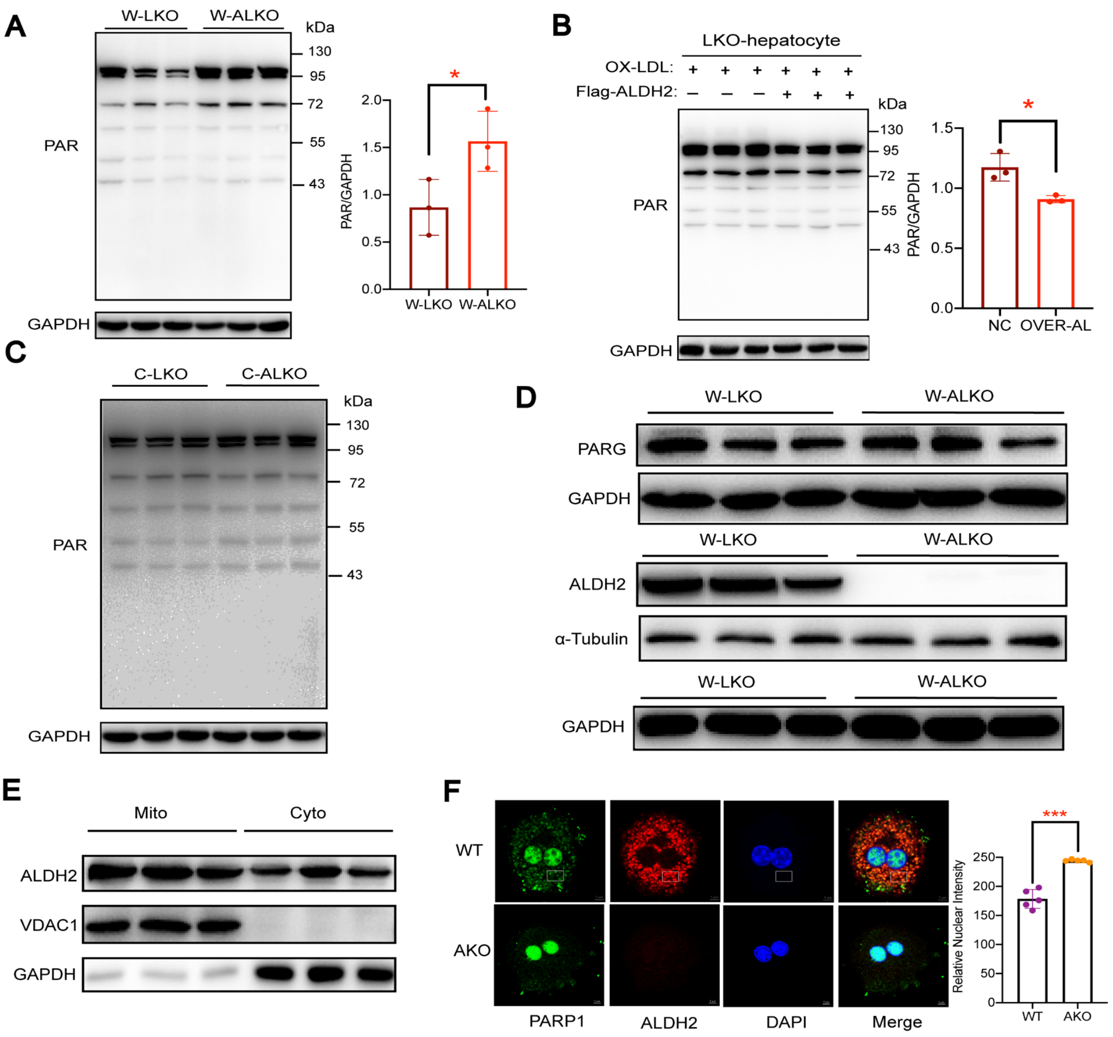

Supplemental Figure 4. ALDH2 modulates poly(ADP-ribosylation) and nuclear translocation of PARP1. (**A**) Western blotting analysis of PAR expression in LKO and ALKO liver tissue at 32nd week (Western Diet for 26 weeks, n=3). (**B**)Western blotting analysis of poly-ADP Ribosylation in overexpressed ALDH2 hepatocytes treated with ox-LDL (50 μg/ml, 16 h, n=3). (**C**) Western blotting analysis of poly-ADP Ribose expression in LKO and ALKO liver tissue at 32nd week (Chow Diet for 26 weeks, n=3). (**D**) Western blotting analysis of PARG expression in LKO and ALKO liver tissue at 32nd week (Western Diet for 26 weeks, n=3). (**E**) Western blot analysis ALDH2 in mitochondria and cytoplasm. (n=3). (**F**) Immunofluorescent results in WT and AKO hepatocyte cells (red, ALDH2; green, PARP1; blue DAPI; Scale bar: 5 μm). ALDH2 inhibit nuclear translocation of PARP1 in WT liver tissue by nuclear separation and quantification (n=5). Immunoprecipitation results of ALDH2 and PARP1 in cytoplasm. Statistical comparisons were made using a 2-tailed Student's t test. All data are mean ± SD. *P < 0.05, **P < 0.01, ***P < 0.001.

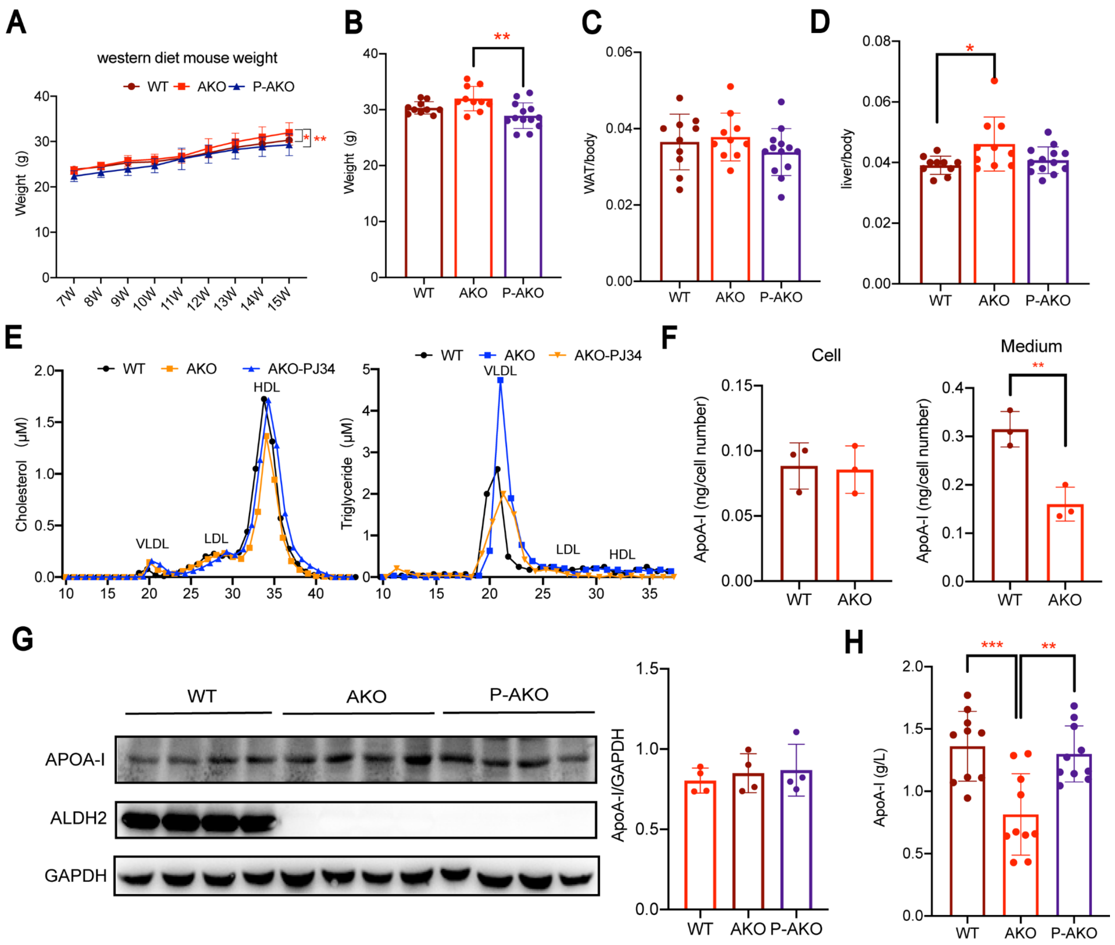

Supplemental Figure 5. PARP1 inhibition by PJ-34 reverses hepatic steatosis and increases HDL-C in AKO mice with Western Diet. (**A**) Body weights in wild type (WT), ALDH2 knockout (AKO) and ALDH2 knockout (AKO) treated with PJ34 (P-AKO). (WT, n=10; AKO, n=10; P-AKO, n=10). (**B-D**) Mouse body weights, percentages of WAT and liver weight/body weights in WT, AKO and P-AKO mice. (WT, n=10; AKO, n=10; P-AKO, n=10). (**E**) FPLC analysis of lipoproteins and triglycerides in serum of WT, AKO and AKO treated with PJ34 liver tissue at 15th week (Western Diet for 8 weeks). (**F**) ApoA-I levels in WT and AKO mouse hepatocytes treated with ox-LDL treatment (50ug/ml, 16h, WT, n=3; AKO, n=3). (**G**) Western blotting analysis of ApoA-I expressions in WD mouse liver tissue. (WT, n=4; AKO, n=4; P-AKO, n=4). (**H**) ApoA-I level in plasma. (WT, n=10; AKO, n=10; P-AKO, n=10). Statistical comparisons were made using One-way ANOVA followed by Student-Newmann-Keuls multiple comparison tests. All data are mean ± SD. *P < 0.05, **P < 0.01.

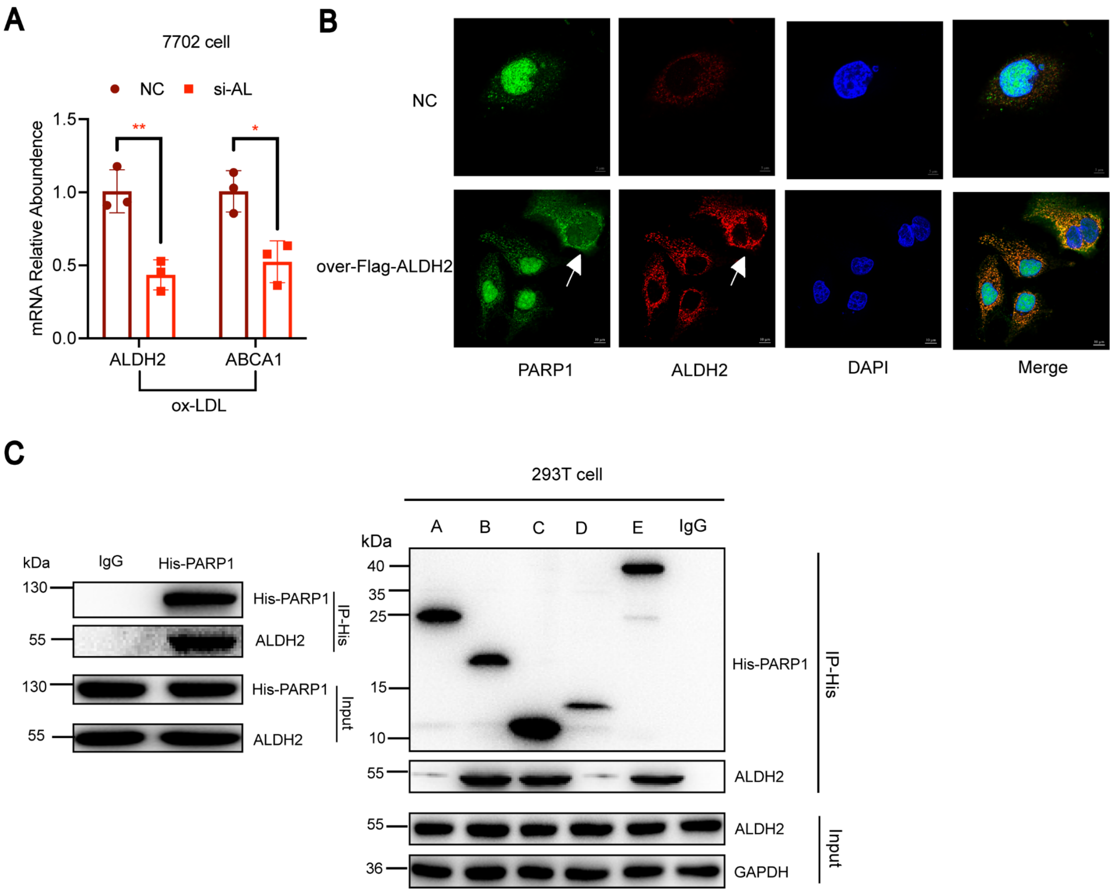

Supplemental Figure 6. ALDH2 modulates PARP1 nuclear translocation and ABCA1 expression. (**A**) mRNA levels of ABCA1 and ALDH2 in HL-7702 cell transfected with ALDH2 siRNA or control siRNA with oxLDL treatment (n = 3) (**B**) Immunofluorescent results in HL-7702 cell transfected with NC and Flag-ALDH2 (red, Flag; green, PARP1; blue, DAPI; Scale bar: 10 μm). (**C**) NLS, BRCT and CHOW DIET mediate the association of PARP1 with ALDH2 in 293T cell. Levels of ALDH2 pulled down by immunoblotting. Statistical comparisons were made using a 2-tailed Student's t test. All data are mean ± SD. *P < 0.05, **P < 0.01.

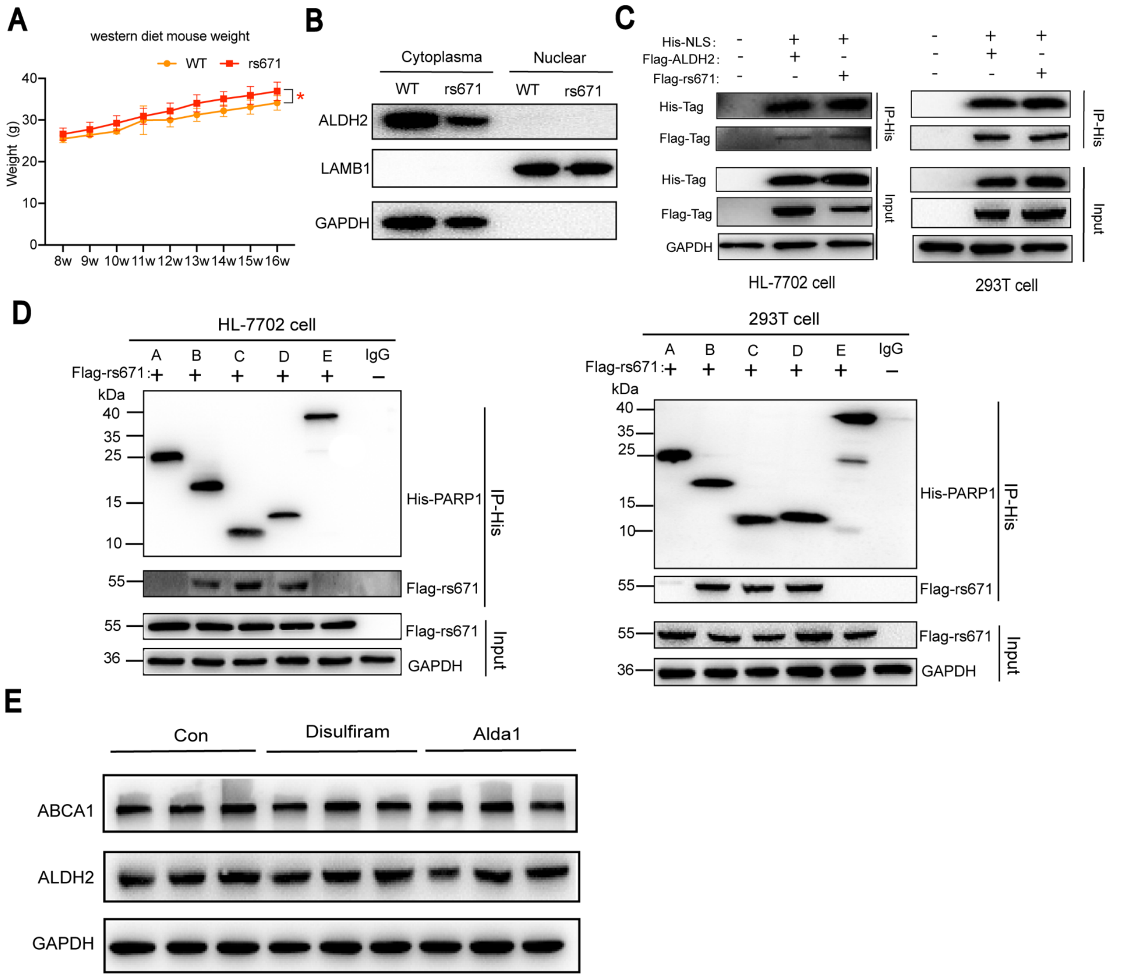

Supplemental Figure 7. ALDH2 rs671 mutation attenuates nuclear translocation of PARP1 *in vitro* and *in vivo*. (**A**) Body weights of WT and 671 mice (Western Diet for 8 weeks started at 8th week of age). (**B**) ALDH2 or ALDH2rs671 distributions in nuclear and cytoplasmic fractions. (**C**) ALDH2 or ALDH2rs671 interact with PARP1 in HL-7702 and 293T cell. (**D**) NLS, BRCT and WGR mediate the association of PARP1 with ALDH2rs671 in HL-7702 and 293T cell. (**E**) Western blotting analysis of ALDH2 and ABCA1expressions in HL-7702 cells treated with Disulfiram (50mM, 16h) or Alda1 (10μM, 16h) (n=3). Statistical comparisons were made using a 2-tailed Student's t test. Statistical comparisons were made using a 2-tailed Student's t test. All data are mean ± SD.
